# Older adults’ coping strategies during the COVID-19 pandemic – a longitudinal mixed-methods study

**DOI:** 10.3389/fpsyg.2023.1209021

**Published:** 2023-09-04

**Authors:** Lydia Kastner, Ulrike Suenkel, Gerhard W. Eschweiler, Theresa Dankowski, Anna-Katharina von Thaler, Christian Mychajliw, Kathrin Brockmann, Walter Maetzler, Daniela Berg, Andreas J. Fallgatter, Sebastian Heinzel, Ansgar Thiel

**Affiliations:** ^1^Institute for Sport Science, Eberhard Karls University of Tübingen, Tübingen, Germany; ^2^Tübingen Center for Mental Health (TüCMH), Department of Psychiatry and Psychotherapy, Tübingen University Hospital, Tübingen, Germany; ^3^German Center for Mental Health (DZPG), Partner Site Tübingen, Tübingen, Germany; ^4^Geriatric Center, Tübingen University Hospital, Tübingen, Germany; ^5^Department of Neurology, University Medical Centre Schleswig-Holstein and Kiel University, Kiel, Germany; ^6^Institute of Medical Informatics and Statistics, University Medical Center Schleswig-Holstein and Kiel University, Kiel, Germany; ^7^Department of Neurodegeneration, Hertie Institute for Clinical Brain Research, University of Tübingen, Tübingen, Germany; ^8^German Center for Neurodegenerative Diseases, University of Tübingen, Tübingen, Germany; ^9^Lead Graduate School and Research Network, Eberhard Karls University of Tübingen, Tübingen, Germany

**Keywords:** COVID-19, coping, Lazarus stress model, psychosocial factors, older adults

## Abstract

**Introduction:**

Older age is a main risk factor for severe COVID-19. In 2020, a broad political debate was initiated as to what extent older adults need special protection and isolation to minimize their risk for SARS-CoV-2 infection. However, isolation might also have indirect negative *psychological* (e.g., loneliness, stress, fear, anxiety, depression) or *physical* (e.g., lack of exercise, missing medical visits) consequences depending on individual strategies and personality traits to cope longitudinally with this crisis.

**Methods:**

To examine the impact of individuals’ coping with the pandemic on mental health, a large sample of 880 older adults of the prospective longitudinal cohort TREND study were surveyed six times about their individual coping strategies in the COVID-19 pandemic between May 2020 (05/2020: *M*_age_ = 72.1, *SD*_age_ = 6.4, Range: 58–91 years) and November 2022 in an open response format. The relevant survey question was: *“What was helpful for you to get through the last months despite the COVID-19 pandemic?* E.g.*, phone calls, going for a walk, or others.”*

**Results and Discussion:**

In total, we obtained 4,561 records containing 20,578 text passages that were coded and assigned to 427 distinct categories on seven levels based on qualitative content analysis using MAXQDA. The results allow new insights into the impact of *personal prerequisites* (e.g., value beliefs, living conditions), the *general evaluation of the pandemic* (e.g., positive, irrelevant, stressful) as well as the *applied coping strategies* (e.g., cognitive, emotional- or problem-focused) to deal with the COVID-19 pandemic by using an adapted Lazarus stress model. Throughout the pandemic emotional-focused as well as problem-focused strategies were the main coping strategies, whereas general beliefs, general living conditions and the evaluation were mentioned less frequently.

## 1. Introduction

In early 2020, the coronavirus (SARS-CoV-2) caused a global health crisis that challenged our health care systems, upended daily life, and led to economic and social upheaval, e.g., lockdowns, quarantine and hygiene regulations (Chen, 2020; Wu and McGoogan, 2020; State of Baden-Württemberg, 2023). Estimates indicate that more than 660 million people worldwide were infected with SARS-CoV-2 by January 2023, of which approximately 6.7 million were fatal (Abab et al., 2022; Johns-Hopkins-University, 2023). Although, most people had only mild to moderate diseases, a substantial minority had a higher risk for severe COVID-19 and adverse health outcomes, such as long- or post-COVID (Abab et al., 2022; Subramanian et al., 2022). Across several countries, mortality rates increased exponentially depending on age and multimorbidity (Bonanad et al., 2020; Chen et al., 2023). Early on, age had been identified as most significant risk factor for severe COVID-19 (Chen et al., 2023) because older adults also have a higher prevalence of chronic diseases such as type 2 diabetes (Zhu et al., 2020; Kompaniyets et al., 2021b), obesity (Kim et al., 2021; Kompaniyets et al., 2021a,b), coronary heart (Lippi and Henry, 2020; Kim et al., 2021), and neurocognitive diseases (Rosenthal et al., 2020; Kim et al., 2021). For instance, it was found that mortality risk increased up to 26% for adults with Alzheimer’s disease and related dementias compared to 2019 (for adults without dementia the risk increased up to 12%, Gilstrap et al., 2022).

This sparked controversial debates about how to deal with an increased vulnerability for COVID-19 in older (or particularly frail) adults. It was claimed that these adults need both a special protection and isolation to minimize their risk of infection and they also need to maintain independence and autonomy to avoid negative *psychological* (e.g., depression, loneliness, anxiety), and *physical* consequences (e.g., lack of exercise, missing medical visits and using negative coping strategies, AgeUK, 2020; Chen, 2020; Promislow and Anderson, 2020; Chen et al., 2023). In Germany, point prevalence for a depressive episode in older adults was 7% (95% CI 4.4–10.6%), and for adults aged 75+ years even 17% (95% CI 9.7–26.1%, Luppa et al., 2012). Despite the expectation that social isolation would lead to a significant health care gap and increased depressive symptoms and loneliness, studies showed that the psychosocial well-being of older adults remained remarkably stable throughout the pandemic (Betsch et al., 2020; Röhr et al., 2020; Minahan et al., 2021; van den Besselaar et al., 2021; Dankowski et al., 2023). Psychological stress, however, was only elevated at the beginning of the pandemic and depended on health status, functional resources, individuals’ participation/activity and living environment (Gaertner et al., 2021). In general, these results might be surprising if we consider the COVID-19 pandemic as a global health crisis in which individuals had to adapt quickly to changes in work, social activities, and quarantine restrictions (Giordano, 2020; Gaertner et al., 2021; Bhattacharjee and Ghosh, 2022). Several studies investigated how older adults coped with stress arising from the pandemic and to what extent individual characteristics, resilience and various coping strategies played a role in this – but only at one particular stage of the pandemic (e.g., Greenwood-Hickman et al., 2021; Bhattacharjee and Ghosh, 2022; Halamová et al., 2022; Iswatun et al., 2022; Kumar et al., 2022; Joseph et al., 2023). *Resilience*, thus, describes the capacity to recover quickly from difficult situations and stressful life events, whereby this in turn depends not only on the psychological prerequisites of the individual but can be considered as a dynamic process allowing positive adaptation in unknown situations such as the COVID-19 pandemic. *Coping* or *coping strategies* describe the active process and specific behavior that protects oneself to avoid negative experiences during stressful life events (Pearlin and Schooler, 1978; Carver et al., 1989; Chen, 2020). Since feelings of stress are a cumulation of thoughts, emotions, and behaviors taking into account internal and external demands, Lazarus and Folkman (1984) described a model in which perceived stress depends on *primary appraisal* of a stimulus as irrelevant, positive or stressful. After the primary appraisal, when a person has determined the relevance and consequences of the stimulus for him- or herself, the *secondary appraisal* involves the evaluation of resources. Therefore, skills the person has acquired in previous stressful situations, self-confidence, but also material resources or social support are needed. The fewer resources a person has to cope with a specific stressful situation, the more intense the stress response will be. These two appraisals do not temporally occur in sequence but may overlap and influence each other and are characterized by person’s perception. After the appraisal is completed, coping occurs. The focus of coping can be on changing the external situation (*problem-oriented coping*), e.g., through the structuring of daily activities or hygiene and protection measures, or on changing internal states and feelings (*emotion-oriented coping*), e.g., through social contacts, self-care, mindfulness. Since the COVID-19 pandemic could be described as a psychological stressful experience, the individual’s cognitive evaluation of the situation (as positive, irrelevant, or stressful) and the resources available to the individual may determine whether coping is necessary at all or the extent to which coping strategies are (or need to be) used.

In the present study, we were interested in how older adults with an increased vulnerability for severe COVID-19 cope with the pandemic-related circumstances over time and how these strategies change over time.

### 1.1. The aim of the present study

Since very little is known in the literature about how vulnerable populations deal with the COVID-19 pandemic longitudinally, we posed the following research question: *How do older adults cope with the COVID-19 pandemic over time?* To answer our research question, we sent questionnaires to a vulnerable population of older adults at continuous intervals over a period of 2.5 years (for more detailed information, see 2.1 research sample). In these, among many other topics, an open-ended question was asked about what the participants experienced as helpful during the pandemic from May 2020 to November 2022. Our first aim was to categorize the responses to the open-ended question (text fragments) using qualitative data analysis and develop a comprehensive category system. Furthermore, in an exploratory quantitative analysis, we aimed to examine associations between coping (strategies) and demographic variables (age, education level), fear of COVID-19, perceived stress, resilience, depression, loneliness, health-related quality of life, and physical (in)activity, as well as gender differences. This proceeding represents a mixed-methods approach.

## 2. Methods and materials

### 2.1. Research sample

The cohort of the present study originates from the prospective longitudinal cohort study “**T**übingen Evaluation of **R**isk Factors for **E**arly Detection of **N**euro**D**egeneration” (TREND), which was initiated in 2009 and is currently in its 5th follow-up (Wave 6). Participants are examined in 2-year intervals. The main purpose of the TREND study is to identify, define, and validate risk factors and prodromal markers for Alzheimer’s and Parkinson’s disease.^1^ For TREND, older adults (aged 50+ years) from the Neckar-Alb and Stuttgart regions (in southern Germany) were recruited, primarily participants with specific prodromal markers for neurodegeneration (“enriched cohort”): lifetime depression, hyposmia, or (probable) REM sleep behavior disorder (RBD). In-depth details about the inclusion and exclusion criteria of TREND can be found in the study protocol (Gaenslen et al., 2014). In addition, participants were included who had previously taken part in another study for early detection of Parkinson’s disease which was population-based (“Prospective evaluation of Risk factors for Idiopathic Parkinson’s Syndrome,” PRIPS; Berg et al., 2010, 2013). A total of 1,201 participants took part in at least one visit of the TREND study. Membership to one or more risk groups (depression, hyposmia, probable RBD) was determined at the first study visit using tests and questionnaires. At the first study visit, 60% of participants had at least one prodromal marker (30% depression, 36% hyposmia, 18% probable REM sleep behavior disorder; for more details see Supplementary Table S1). Furthermore, 14% had first-degree relatives with Parkinson’s disease, and 31% with dementia, and participants thus had an increased risk of developing the diseases. The study follows the guidelines for good scientific practice at the University of Tübingen (Germany), the Declaration of Helsinki (1964) and its later amendments and was approved by the local ethics committee of the University Hospital Tübingen (No 90/2009BO2). All participants gave their written informed consent to participate in the study.

Due to the COVID-19 pandemic, lockdown and hygiene recommendations of the regional government and the Robert Koch Institute, the regular TREND data collection had to be paused immediately in March 2020 to minimize our participants’ risk of infection with SARS-CoV-2 (Governmental Regulation of the State of 266 Baden-Württemberg from 03/17/2020, CoronaVO). In the following, the research question arose how our cohort with increased vulnerability (older age, increased risk for neurodegenerative diseases) would cope with the pandemic longitudinally, especially the protective measures such as self-isolation and general restrictions. As it is known from the literature, adults who are at increased risk for dementia are also at increased risk for severe COVID-19 progression and accelerated cognitive decline (Chen et al., 2023). To investigate the impact of the COVID-19 pandemic on our cohort, six Corona questionnaires (Coro-Q, in the following referred to as Coro-Q1 to Coro-Q6 in Tables/Figures, e.g., Coro-Q1 means Corona questionnaire No. 1) on general, health- and pandemic-related aspects were sent to the participants via post and later also online. Eight hundred and eighty participants of the TREND cohort were willing to take part in these COVID-19 pandemic related questionnaires at least once (mean age in May 2020: *M* = 72.1, *SD* = 6.4, Range: 58–91 years; 48.3% females, years of education: Mdn = 14, IQR: 12–16 years; for demographics of each questionnaire round see Table 1). The first questionnaire was sent by post in May 2020, followed by five more questionnaires approximately every 6 months (paper or online questionnaires, depending on participants’ preference). The response rates for each questionnaire were > 80%. Participants did not reach any financial or other benefit of the participation in the pandemic-related questionnaire study. However, it should be noted that most participants had been taking part in TREND for over 10 years at the onset of the pandemic, and many participants had developed a strong commitment to the study and a bond with the longstanding, consistent study team over time. This may have contributed to the exceptionally high response rates. Table 1 provides an overview of the questionnaire rounds [Coro-Q1 to Coro-Q6, and demographic characteristics of the respondents (*N*_total_ = 4,561)]. Of the 880 participants who completed at least one Corona questionnaire (Coro-Q), 56% had at least one prodromal marker for neurodegeneration (29% depression, 31% hyposmia, 17% probable REM sleep behavior disorder); 14% had first degree relatives with Parkinson’s disease and 35% with dementia (for exact numbers and percentages for each risk group and combination of prodromal markers see Supplementary Table S1).

**Table 1:** Questionnaire rounds (Coro-Q1 to Coro-Q6) and demographic characteristics of the participants.

Questionnaire round		3-Month Coro-Q1	8-Month Coro-Q2	14-Month Coro-Q3	20-Month Coro-Q4	25-Month Coro-Q5	32-Month Coro-Q6
Response date (median [month/year])		June-20	November-20	May-21	November-21	April-22	November-22
Number of surveys sent	Total (*n*)	932	909	899	880	868	854
	Paper (*n*, %)	932 (100%)	909 (100%)	540 (60%)	519 (59%)	495 (57%)	480 (56%)
	Online (*n*, %)	–	–	359 (40%)	361 (41%)	373 (43%)	374 (44%)
Number of respondents	Total (*n*, %)	774 (83%)	780 (86%)	796 (89%)	759 (86%)	746 (86%)	706 (83%)
	Paper (*n*, %)	774 (83%)	780 (86%)	445 (82%)	412 (79%)	392 (79%)	367 (76%)
	Online (*n*, %)	–	–	351 (98%)	347 (96%)	354 (95%)	339 (91%)
At least one codable text fragment on the coping strategies question^1^	*(n, %)*	667 (86.2%)	694 (89.0%)	709 (89.1%)	653 (86.0%)	635 (85.1%)	610 (86.4%)
Age (yrs)	*M*	72.3	72.5	73.0	73.5	73.8	74.3
	*SD*	6.3	6.3	6.2	6.3	6.2	6.2
	Range	58–91	59–91	59–92	60–92	60–93	61–93
Females	*n* (%)	367 (47.5%)	377 (48.3%)	380 (47.7%)	364 (48.0%)	354 (47.5%)	333 (47.2%)
Education (yrs)	Median	14.0	14.0	14.0	14.0	14.0	14.0
	IQR	12–16	12–16	12–16	13–16	13–16	13–16
	Range	9–21	9–21	9–21	9–22	9–22	9–22

IQR, interquartile range; M, Mean; SD, standard deviation; yrs, years. ^1^Question: “What was helpful for you to get through the last months despite the COVID-19 pandemic? E.g., phone calls, going for a walk, or others.”

As of June 2023, TREND has a total of 77 subjects who have developed a severe neurodegenerative disease (Parkinson’s disease, dementia, or other); of these, 27 have completed a Corona questionnaire at least once (14 subjects diagnosed with Parkinson’s disease, 11 diagnosed with dementia, one diagnosed with progressive generalized chorea, one with amyotrophic lateral sclerosis), with 13 of these participants receiving their diagnosis during the course of the pandemic (2021 to 2023) (four Parkinson’s disease, nine dementia). Overall, the subcohort of TREND that completed at least one Corona questionnaire contains 3% subjects with a severe neurodegenerative disease.

### 2.2. Questionnaires

From May 2020 to November 2022, more than 800 older adults were surveyed six times (Coro-Q1 to Coro-Q6) at 6-month intervals about their fear of getting COVID-19, depression, perceived stress, loneliness, resilience, health-related quality of life, and level of physical (in)activity. Table 2 shows selected material used in the questionnaire rounds. At the end of each of the abovementioned six Coro-Q questionnaires, there was a question about personal coping with the COVID-19 pandemic in an open-ended response format: *“What was helpful for you to get through the last months despite the COVID-19 pandemic? (E.g., phone calls, going for a walk, or others).”* Because the active subset of the TREND cohort at the beginning of the pandemic still consisted of more than 900 subjects, we were unable to interview each participant in person using semi-structured interviews. For this reason, we had to rely on postal or online questionnaires. In total, we obtained 4,561 records in the six biannual questionnaire rounds. An impressive and unique set of qualitative longitudinal data on the pandemic, health-related and psychosocial factors of older adults’ personal coping with the COVID-19 pandemic was collected over a 2.5-year period. Study data were collected and managed using REDCap electronic data capture tools hosted at the University of Tübingen (Harris et al., 2009).

**Table 2:** Used material per questionnaire round (May 2020 to November 2022).

Questionnaire	Description
Fear of COVID-19	Fear of COVID-19 was measured on a scale from 0 (*no fear at all*) to 10 (*very much fear*).
Depression	To measure severity of depression, the *Becks Depression Inventory* (BDI) was used as self-report questionnaire. It was developed in the USA in 1961, revised in 1978 (Beck et al., 1961, 1987); the latest German translation and validation for the BDI-I (Hautzinger et al., 1996). Since 1996, there has been a newer version adapted to DSM-IV (BDI-II, Beck et al., 1996) for which the latest German translation and validation used in TREND is from 2009 (Hautzinger et al., 2009). Participants had to choose one of four statements which they mostly described their feelings and behavior in the last 2 weeks. Thereby, 0–13 scores indicate minimal depression, 14–19 mild depression, 20–28 moderate depression, and 29–63 severe depression. Scores ≥14 are referred to as clinically relevant depression.
Perceived Stress	Stress was assessed with 10 items using the *Perceived Stress Scale* (PSS, 58). Participants were asked how often they felt stressed in the last month (example-item: *‘In the last month, how often have you been upset because something unexpected happened?’*, answer options: *never, almost never, sometimes, quite often, very often*). The total score ranges from 0 (*no perceived stress*) to 40 points (*very strong perceived stress*).
Resilience	To measure resilience, we used the *Brief Resilience Scale* (BRS, Chmitorz et al., 2018) consisting of 6 items, e.g., *‘I tend to recover quickly after difficult times’* with response options on a 5-point-likert scale from *‘strongly disagree’* to *‘strongly agree’*. Resilience scores range from 1 (*low resilience*) to 5 (*high resilience*).
Loneliness	Since loneliness is associated with depression (Klein et al., 2016), we used a 6-item questionnaire (Gierveld and Tilburg, 2006) to measure overall loneliness. Participants were asked to indicate on a 4-point Likert scale how much they agree with the statements personally (*not at all true* to *true exactly*) in the last 3 months (example-item: *‘I miss people who make me feel good’*). Total scores range from 0 (*not lonely at all*) to 6 (*very lonely*).
Health-related quality of life	To measure participants’ health-related quality of life, the EQ-5D-5L (Herdman et al., 2011; Feng et al., 2021) visual analog self-report scale was used with endpoints labeled *‘The worst health you can imagine’* (0) and *‘The best health you can imagine’* (100 scores).
Physical (in)activity	Since there is a strong association between depression and physical (in)activity (Mura and Carta, 2013), we decided to analyze physical (in)activity as ordinal data of *‘no activity’*, *‘< 1 h (hrs)/week’*, *‘1–2 h/week’*, ‘*2–4 h/week*’, and *‘> 4 h of physical activity per week’* with increased heart-rate or sweating using a standardized questionnaire (Thefeld et al., 1999).
Coping strategies	Since we were interested in how participants were coping with the COVID-19 pandemic, we used an open response format to answer the question: *“What was helpful for you to get through the last months despite the COVID-19 pandemic?* E.g.*, phone calls, going for a walk, or others.”*

### 2.3. Mixed-methods approach

The core of this article is a mixed-methods analysis to answer our research question on how older adults with increased vulnerability for severe COVID-19 (Chen et al., 2023) deal with the pandemic situation longitudinally (*cf.* mixed-methods or hybrid approach, Hussy et al., 2010).

First, a *qualitative* content analysis was conducted on the textual information that participants were asked to provide at the end of the questionnaire by indicating what they found helpful for coping with the pandemic. In response to the open-ended question *“What was helpful for you to get through the last months despite the COVID-19 pandemic? (E.g., phone calls, going for a walk, or others),”* we received answers in text format. These ranged from one-word answers through lists to shorter or longer text fragments (in complete sentences). For organizing and coding the text material, we used the qualitative analysis software MAXQDA (VERBI-Software, 2021). MAXQDA is a computer-assisted qualitative data analysis software (CAQDAS) designed to assist researchers in managing and analyzing qualitative and mixed-methods data, provided in a range of tools to facilitate the organization, coding, analysis, and visualization of data. As method, we used the widely used and established qualitative content analysis according to Mayring (2015, 2020), which enabled us to analyze the text material (summarizing, explicating, structuring), form categories, and combine two approaches: (1) *inductive category development* (“bottom-up approach”) and (2) *deductive category application* (“top–down approach”). Accordingly, in a first step, we inductively coded the text material and derived a preliminary category system. In long team and expert discussions, it turned out that the code system so far was insufficient regarding many text passages that described for instance general value beliefs or the evaluation of the pandemic situation and did not directly represent coping strategies (e.g., *“faith in god,”* “*having a garden*”). Due to this problem, some text passages from our participants could not be logically integrated into our category system. At this point, as it is also part of the method according to Mayring, we added the deductive approach and started searching for definitions and classifications of coping and coping strategies (for an overview see Skinner et al., 2003). Thereby, we encountered Lazarus and Folkman’s transactional stress model (Lazarus and Folkman, 1984). This model offered a solution for handling text passages about general value beliefs or the evaluation of the pandemic situation. Thus, in a second step, we restructured our category system deductively using the theoretical framework of Lazarus and Folkman by considering the pandemic situation as stress. Overall, after inductive category formation with recourse to the transactional stress model (Lazarus and Folkman, 1984), we were able to deductively classify all text material in the sample into a logical and comprehensive category system.

Once the final category system was defined, we were able to calculate the numbers for each category and each participant for all six questionnaire rounds. In a *quantitative* exploratory analysis, we investigated how our main categories correlate with demographic variables (age, years of education), depression, perceived stress, resilience, loneliness, health-related quality of life, physical (in)activity, and examined gender differences (Herrera-Añazco et al., 2022; Peyer et al., 2022). Kendall’s tau B was used for correlations and Mann–Whitney U tests for group comparisons because of the skewness of the data. Quantitative data analysis was performed using the software SPSS version 29.0 (IBM-Corp, 2021).

## 3. Results

### 3.1. Results of the mixed-methods analysis

Through the qualitative content analysis in MAXQDA and deductively using an adapted/extended Lazarus stress model, a total of 20,578 text passages could be coded and 427 categories could be formed which are organized on seven hierarchic levels, with level 1 representing the highest and level 7 the lowest (for details, see Supplementary Table S1). In this article, we use the term “categories” to refer to the related content that has been organized hierarchically. Categories at higher levels represent supercategories and stand for a topic area (e.g., problem-focused strategies) to which further categories are subordinated (e.g., structuring everyday life). The more detailed a topic is represented in the category system, the more levels that topic has. The categories are mutually exclusive and the representation in Supplementary Table S2 is not cumulative, since it was possible that very generally formulated text fragments were sorted directly into a higher level without belonging to one of the subordinate levels. However, the category system can be aggregated at each level by cumulating the numbers of the lower levels and adding them to the numbers of the higher levels.

We obtained six main categories on level 1 (C1–C6): (1) C1: *General Beliefs (concepts/values/convictions)* (*N* = 234), (2) C2: *General Living Conditions (material/financial/social)* (*N* = 1,252), (3) C3: *General Evaluation of the Situation (meta-reflection* as positive, irrelevant, or stressful*)* (*N* = 863), (4) C4: *Problem-focused Strategies* (*N* = 9,925), (5) C5: *Emotion-focused Strategies* (*N* = 8,049), (6) C6: *Cognitive Strategies (reactive)* (*N* = 255). Thereby, C1 and C2 describe the general prerequisites that a person possesses in terms of values and material/financial/social resources, whereas C3 represents the general evaluation of the situation in the form of the primary appraisal as positive, irrelevant, or stressful. This is followed by the secondary appraisal, considering whether sufficient resources are available to deal with the problem. C4–C6 represent the specific coping strategies in dealing with the problem, where either the external situation is to be changed by problem-focused coping (e.g., daily structuring) or the internal attitude with respect to emotions (e.g., by emotion regulation through eating, social contacts) or cognitions (e.g., by distraction, attitude change). A definition of the 6 main categories (and subcategories up to level 3) and examples can be found in Table 3. For details on the distribution of the numbers of the main categories among the 6 questionnaire rounds, see Table 4 (a more detailed table with all 427 categories can be found in the Supplementary Table S1) and for relative frequencies of how often each category was used, see Table 5. On average, across all six questionnaire rounds, participants most frequently used problem-focused coping strategies in dealing with the pandemic {Coro-Q1 (*Median* [IQR]): 2 [0;4]; Coro-Q2: 2 [1;4]; Coro-Q3: 2 [1;3]; Coro-Q4: 2 [1;3]; Coro-Q5: 2 [0;3]; Coro-Q6: 2 [0;3], *cf.* Table 4}. Few participants reported their general value beliefs, general life circumstances, their general evaluation of the pandemic or cognitive strategies (see Tables 4, 5).

**Table 3:** A definition of the 6 main categories (subcategories up to level 3) and participants’ examples.

Level 1 Level 2 Level 3	Definition	Examples
C1: General Beliefs (concepts/values/convictions)		
Life Attitude and Experience Positive Thinking/Optimism Self-motivation Resilience Joy of Life Faith/Spirituality Own Health Status Introverted/used to Being Alone	General values are values/ways of thinking that the person brings with him/her based on life experiences and personality traits.	*“Perhaps also my entire attitude to life.”* [2_7367] *“Positive thinking”* [1_1220], *“I always see the half-full glass and not the half-empty glass!”* [1_7272] *“My will not to give up*” [6_7068], *“have enough initiative”* [6_7145] *“My own adaptability, to put things into perspective and take them as they are”* [2_9068] *“Laugh a lot”* [4_7483] *“Who counts on God, saves worries”* [1_7028], *“grateful to be connected with Jesus”* [2_1217] *“Physical and mental health”* [4_1359], *“the end of my depression in March 2020”* [4_7439] *“I can be well alone”* [6_7522], *“Being more of a loner, I had little problem because of the contact restrictions”* [8_7215]
C2: General Living Conditions (material/financial/social)		
Good Health System/Medical Care Not Being/Living Alone Autonomy/Independence/Self-reliance Be Retired Financial Security Housing Situation Good Living Environment Living Atmosphere (apartment, feeling comfortable at home) Specific Housing Situation/Amenities Mobility Bicycle/Walking Instead of Public Transport Mobility by Public Transport Mobility by Own Means of Transport	General living conditions are understood as the material (housing situation), financial (pension, financially secure) and social (mobility) conditions that a person brings with him/her.	*“happy to live in Germany, and knowing should one become infected the medical care is very good”* [1_1175] *“not living alone in the household”* [6_1453] *“That I can take care of myself without help”* [8_7274], *“I am autonomous, can take care of myself”* [2_7162] *“As a pensioner, you have almost no restrictions.”* [2_1460], *“Since I am a pensioner, I could organize my day as I wanted”* [1_1691] *“financial independence”* [1_1366], *“No financial worries”* [2_7002] *“home environment”* [6_7128], *“my home gives me a feeling of security”* [1_1454] *“optimal residential location”* [1_7015], *“Ideal location of the apartment: quiet, garden, all routes within walking distance”* [2_7015] *“Feeling good at home within your own “4 walls.””* [6_1250] *“I have my own house with a large garden, due to which I can withdraw and occupy myself in and around the house”* [8_7350] *“Doing the shopping on foot”* [1_1529] *“Use public transportation to get out of the house”* [4_7253] *“own car (=less risk of infection)”* [2_1056], *“Travel with camper”* [6_7056]
C3: General Evaluation of the Situation (meta-reflection)		
Positive Better/Longer Sleep (Quality) Having More Time No Boredom Freedom from Obligations/Appointments Less People Outside (Intensive) Connection With Others Irrelevant Pandemic not as Threatening as Before There were more Important Things than the Pandemic No Restriction/Change due to Pandemic Hardly any Restriction/Change due to Pandemic No special Support needed during the Pandemic Do not miss Personal Contacts No Fear (of Corona/Infection) Joy/Relief about Relaxation of Corona Rules Stressful Damage/Loss Threat Challenge	The general evaluation of the situation describes the meta-reflection of the current situation, which Lazarus and Folkman (1984) defined as primary appraisal as positive, irrelevant or stressful. This evaluation is the precondition for further coping strategies.	*“shut down” = “+” for “private”* [1_7633] *“Due to the rest, better sleep”* [1_7260] *“more time for own family”* [1_1211] *“I never get bored, I always find something to keep me busy”* [2_7181] *“Exemption from any obligations and deadlines”* [1_7015] *“Deserted walking paths almost on the doorstep”* [2_7233] *“Engage more intensively with familiar people”* [1_7008] *“The pandemic was no longer perceived as threatening as it was in 2020 and 2021”* [8_7629] *“For the last few months, Corona has been less of a concern to me than the heat”* [8_1714] *“Just continue to live normal life with the rules”* [1_1423] *“Life goes on”* [2_7190] *“My daily routine has barely changed”* [1_7034] *“I do not miss personal contact.”* [4_7646] *“I am not afraid of Corona”*[1_7367] *“The relaxations of the regulations”* [6_1621] *“What is missing: the training in the gym, the direct contact with friends, acquaintances, also authorities, etc, the free travel, it is easier to describe that than all the positive things that remained”* [1_7283] *“Corona is almost not on my mind at the moment, unlike the Ukraine war.”*[7_7530] *“biggest nuisance the vaccination chaos!”* [4_1427]
C4: Problem-focused strategies		
Active engagement with the Corona Pandemic Information Gathering Compliant Behavior Caution and Consideration Talking to others about Corona Critical Questioning of Corona Trust in Government/Politics/Authorities/Measures Active Criticism of and Resistance to Corona Rules Knowledge about the Health Status of Relatives No/Less Preoccupation with Pandemic Structuring Everyday Life Everyday Tasks (Leisure) Activities Receiving Instrumental Social Support Cleaning Help Care Service Relatives Live in the same House Get Help with Errands Ask for Help Know about possible Support from Others	Problem-focused strategies are used when the primary appraisal requires action in the sense of coping strategies. One possible strategy is to focus on the problem (in this case, the pandemic) by means of active strategies such as structuring the day or actively dealing with the Corona pandemic (e.g., critical questioning, gathering information).	*“obtain information”* [7_1056] *“be at home a lot”* [2_1816] *“careful contact with the neighbors”* [2_7350] *“Talking to my friends about vaccination”* [4_1547] *“Critical questioning of government corona policies”*[4_1326] *“Professional management of the authorities”* [1_7049] *“Participation in demos against Corona policies”* [7_7684] *“Since my family and I are healthy, I was not very worried”* [1_1314] *“Not watching the news, reading little to no newspapers”* [8_7324] *“Structured day - plan day and take and work on tasks/things”* [1_1674] *“Various challenges (house, garden, financing, etc.)”* [4_7218] *“Cultural participation via TV, radio*, e.g.*, also outdoor cultural offerings”* [1_1154] *“My family and friends supported me, I wanted for nothing”* [4_7547] *“Cleaning help me partly, as far as necessary”* [2_7273] *“Help from care service in caring for my husband with Parkinson’s disease”[4_7315]* *“My son’s family living in the same house (2 separate apartments, 2 children 4 and 2 years old)”* [2_7543] *“Food brought by the children and the neighborhood assistance”* [1_7222] *“I also learned to ask for help”* [6_1714] *“The knowledge that if necessary someone is there to “help”* [8_1710]
C5: Emotion-focused strategies		
Maintain/Seek out Social Contacts Type/Size of Social Contacts Social Form of Interaction Receiving Emotional Social Support Self-care Care for Physical Well-Being Mindfulness Diary/Journaling Enjoy Season/Nature Religion/Fait Related Activities Prayer Church Services Community with other Believers Church Engagement Reading the Bible Resort to Unhealthy Coping Strategies Frustration Eating Unhealthy Diet Drink (too much) Alcohol	Another possibility when the primary appraisal requires action is emotion-focused coping, which allows dealing with the situation by regulating emotions (specifically in the pandemic situation: by staying in contact with other people, self-care, or religion).	*“In general, it was very helpful to have regular contacts with other people”* [1_7416] *“Phone calls with the children”* [1_1453] *“Conversations, discussions”*[1_1356] *“Lovingly concerned and helpful children and grandchildren”* [1_1205] *“Good relationship with myself that carries, regardless of external changes”* [1_7319] *“Visit to a salt hall for inhalation”* [2_7703] *“mindfulness”* [4_9061] *“I made daily notes about the day, as a corona diary”* [1_9062] *“Enjoy the nature”*[1_1781] *“Worship songs”* [1_1491] *“Turn to God in prayer”*[8_7134] *“Possibility to attend church services”*[2_7348] *“Meetings with other believers”* [4_7491] *“church commitment”* [4_7241] *“Read God’s Word (Bible) daily”* [2_7077] *“(too much) chocolate”* [2_9095] *“I have the feeling of living under a glass bell jar with no prospect of improvement. In the meantime, I have become a frustration eater (chocolate)”* [6_1,171] *“I bought food that just caught my fancy - it wasn’t exactly healthy, but it tasted good to me”* [1_7343] *“In the evening I drink a beer with my neighbors (Corona distance)”*[1_7350]
C6: Cognitive strategies (reactive)		
(Re)assessment of the Situation Social Comparison/Relativization (Change) Attitude/Basic Mindset Stay Calm Focus on the Positive Focus on Others Distraction Hope Give up Hope Anticipation of the Time after the Pandemic Hope for Normality soon Hope for Vaccine Planning Plan the Future Plan a Move Plan Vacation/a Trip	Another strategy after the primary appraisal is the cognitive strategies, which include reappraisal of the situation, but also distraction, hope, or planning activities by attention shifting.	*“Many things that were always so important before became increasingly relative”* [2_7272] *“Compared to other countries, we are doing very well here”* [1_1140] *“No time for musings that do not go far anyway”* [4_7323] *“keep calm”* [1_9014] *“Gratitude for being able to look back on a “rich,” colorful life”* [1_7348] *“Helping others*, e.g.*, listening, comforting* etc.*”* [7_9054] *“All activities that distract one from the topic of pandemic (not only Corona)”* [8_1253] *“Hope for a good ending*!!*!”* [7_1,186] *“At the beginning of the pandemic, I found it easier for me to deal with the changes, currently it is somehow more stressful because it is not foreseeable how long this situation will last”* [2_7171] *“Joy of sporting activities allowed again, joy of planned excursions and vacations”* [4_9078] *“The hope for normality soon”* [1_1018] *“The belief that a vaccine will be found quickly”* [1_7108] *“Organize events”* [7_1794] *“More time to think about the future”* [2_7040] *“To prepare and organize my move from my house to an apartment”* [2_7175] *“Travel planning for the time after Covid-19”* [2_7167]

**Table 4:** Descriptive statistics of the six main categories: an overview of how often participants named each strategy.

	Coro-Q1 Jun-20*N* = 774			Coro-Q2 Nov-20*N* = 780			Coro-Q3 May-21*N* = 796			Coro-Q4 Nov-21*N* = 759			Coro-Q5 Apr-22*N* = 746			Coro-Q6 Nov-22*N* = 706		
	Mean (SD)	Median [IQR]	Range	Mean (SD)	Median [IQR]	Range	Mean (SD)	Median [IQR]	Range	Mean (SD)	Median [IQR]	Range	Mean (SD)	Median [IQR]	Range	Mean (SD)	Median [IQR]	Range
C1: general beliefs	0.1 (±0.2)	0 [0;0]	0–2	0.1 (±0.3)	0 [0;0]	0–3	0.0 (±0.2)	0 [0;0]	0–2	0.1 (±0.3)	0 [0;0]	0–3	0.0 (±0.2)	0 [0;0]	0–2	0.0 (±0.2)	0 [0;0]	0–2
C2: general living conditions	0.4 (±0.8)	0 [0;1]	0–5	0.3 (±0.7)	0 [0;1]	0–7	0.3 (±0.6)	0 [0;0]	0–4	0.2 (±0.5)	0 [0;0]	0–4	0.2 (±0.5)	0 [0;0]	0–3	0.2 (±0.5)	0 [0;0]	0–5
C3: general evaluation of the situation	0.3 (±0.8)	0 [0;0]	0–6	0.2 (±0.6)	0 [0;0]	0–4	0.1 (±0.5)	0 [0;0]	0–4	0.1 (±0.4)	0 [0;0]	0–4	0.2 (±0.5)	0 [0;0]	0–4	0.2 (±0.5)	0 [0;0]	0–4
C3.1: positive	0.1 (±0.4)	0 [0;0]	0–4	0.1 (±0.3)	0 [0;0]	0–4	0.0 (±0.1)	0 [0;0]	0–1	0.0 (±0.1)	0 [0;0]	0–1	0.0 (±0.1)	0 [0;0]	0–2	0.0 (±0.2)	0 [0;0]	0–3
C3.2: irrelevant	0.1 (±0.3)	0 [0;0]	0–2	0.1 (±0.3)	0 [0;0]	0–2	0.0 (±0.2)	0 [0;0]	0–1	0.0 (±0.2)	0 [0;0]	0–2	0.1 (±0.3)	0 [0;0]	0–2	0.1 (±0.3)	0 [0;0]	0–2
C3.3: stressful	0.1 (±0.4)	0 [0;0]	0–4	0.1 (±0.4)	0 [0;0]	0–3	0.1 (±0.4)	0 [0;0]	0–4	0.1 (±0.4)	0 [0;0]	0–4	0.1 (±0.4)	0 [0;0]	0–4	0.0 (±0.2)	0 [0;0]	0–2
C4: problem-focused strategies	2.3 (±2.2)	2 [0;4]	0–13	2.3 (±2.2)	2 [1;4]	0–12	2.3 (±2.1)	2 [1;3]	0–12	2.0 (±1.9)	2 [1;3]	0–15	2.0 (±1.9)	2 [0;3]	0–11	2.1 (±2.1)	2 [0;3]	0–14
C5: emotion-focused strategies	1.8 (±2.1)	1 [0;3]	0–11	1.9 (±2.0)	1 [0;3]	0–9	1.9 (±1.9)	1 [0;3]	0–8	1.8 (±1.9)	1 [0;3]	0–11	1.5 (±1.8)	1 [0;2]	0–12	1.7 (±1.9)	1 [0;3]	0–8
C6: cognitive strategies (reactive)	0.1 (±0.4)	0 [0;0]	0–5	0.1 (±0.3)	0 [0;0]	0–3	0.0 (±0.2)	0 [0;0]	0–2	0.0 (±0.2)	0 [0;0]	0–2	0.0 (±0.3)	0 [0;0]	0–5	0.0 (±0.2)	0 [0;0]	0–2
Total number of categories	5.1 (±4.1)	4 [2;8]	0–20	5.0 (±3.8)	4 [2;7]	0–18	4.7 (±3.5)	4 [2;7]	0–20	4.2 (±3.3)	4 [2;6]	0–19	3.9 (±3.3)	3 [1;6]	0–19	4.2 (±3.4)	4 [2;6]	0–20

**Table 5:** All participants who ever took part in one of the questionnaires (*N* = 880).

	Coro-Q1	Coro-Q2	Coro-Q3	Coro-Q4	Coro-Q5	Coro-Q6	Total
	*N* = 774	*N* = 780	*N* = 796	*N* = 759	*N* = 746	*N* = 705	
C1: general beliefs	45 (1.2%)	62 (1.6%)	39 (1.0%)	38 (1.2%)	25 (0.9%)	25 (0.9%)	234 (1.1%)
C2: general living conditions	327 (8.4%)	263 (6.8%)	241 (6.4%)	156 (4.8%)	137 (4.8%)	127 (4.3%)	1,251 (6.1%)
C3: general evaluation of the situation	234 (6.0%)	175 (4.5%)	111 (3.0%)	93 (2.9%)	136 (4.7%)	114 (3.9%)	863 (4.2%)
C4: problem-focused strategies	1810 (46.3%)	1827 (47.0%)	1828 (48.8%)	1,526 (47.4%)	1,464 (50.8%)	1,464 (50.1%)	9,919 (48.2%)
C5: emotion-focused strategies	1,425 (36.4%)	1,493 (38.4%)	1,490 (39.8%)	1,382 (42.9%)	1,087 (37.7%)	1,171 (40.0%)	8,048 (39.1%)
C6: cognitive strategies (reactive)	70 (1.8%)	64 (1.6%)	37 (1.0%)	27 (0.8%)	34 (1.2%)	23 (0.8%)	255 (1.2%)
Total	3,911 (100%)	3,884 (100%)	3,746 (100%)	3,222 (100%)	2,883 (100%)	2,924 (100%)	20,570 (100%)

All available records (*N* = 4,561).

**Figure 1:**
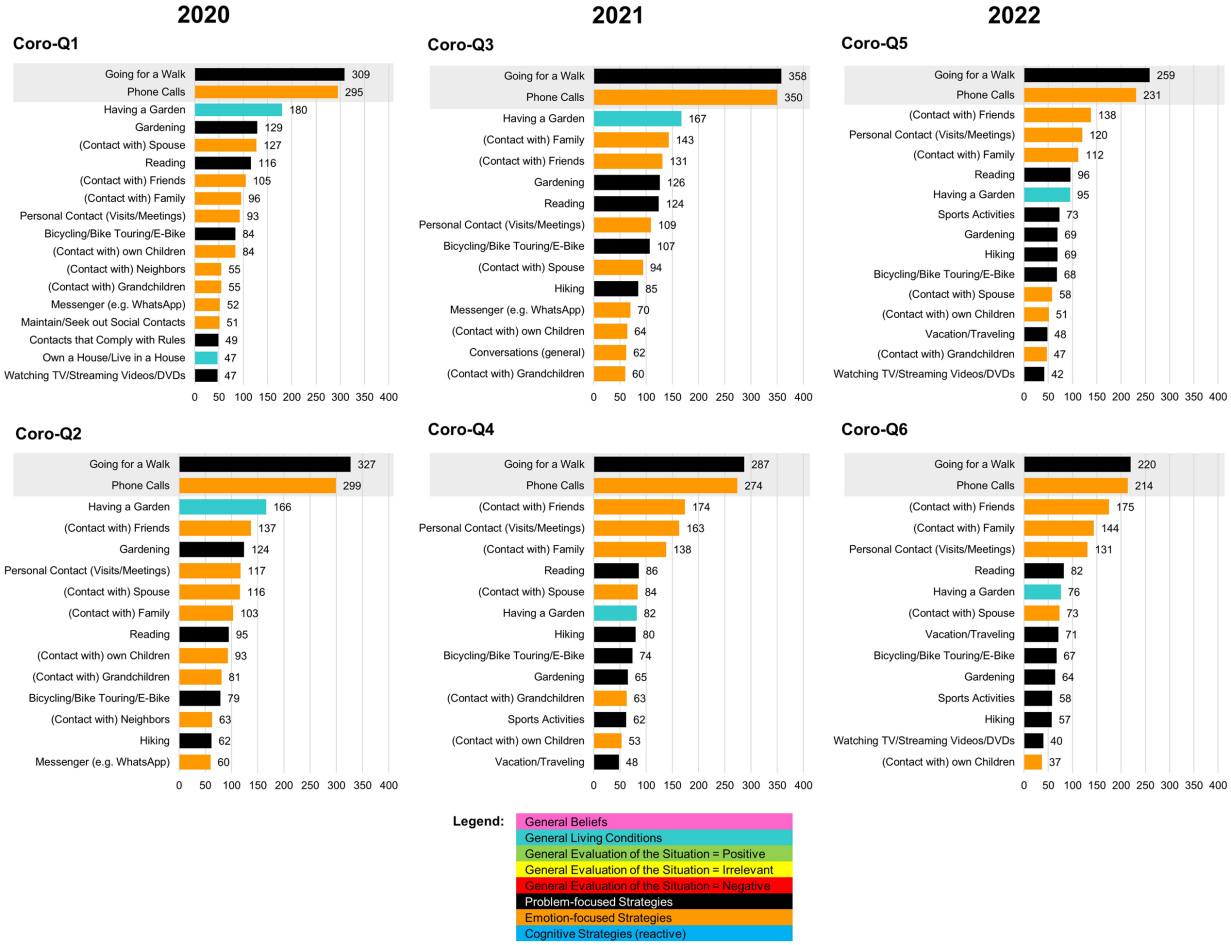
Overview of the Top 15 mentioned items for coping with the COVID-19 pandemic. Participants had to answer the question *“What was helpful for you to get through the last months despite the COVID-19 pandemic?* E.g., *phone calls, going for a walk, or others*”.

In further analyses, we were interested in what was most frequently mentioned by the participants. Therefore, for each round of questionnaires, the 15 most frequently mentioned categories were identified from the 427 categories (Figure 1). In 2020, among the top 15, going for a walk (top 1) and phone calls (top 2), as well as having a garden (top 3), were most frequently mentioned. In addition, many emotion-focused strategies were mentioned, such as contact with a spouse, friends, family, and neighbors. In 2021, going for a walk and phone calls continued to be among the top 3, with more problem-focused strategies added, such as bicycling, gardening, sports activities, or traveling, which was found again in a relatively similar manner in 2022. Across all rounds, emotion-focused strategies (social contact to individuals online or personally) were consistently listed. However, it should be noted that the top 15 are probably skewed by the fact that examples were suggested in the open-ended question. For “general living conditions,” two items reached the top 15 at the beginning of the pandemic, namely “having a garden” and “own a house/live in a house.” During the pandemic, “having a garden” lost some ranks, but remained consistently among the top 15 mentions. In contrast, general beliefs and evaluation of the situation were mentioned less frequently, so that they do not appear in the top 15 (see Discussion). For a more detailed overview of the top 15, see Figure 1.

### 3.2. Results of the exploratory analysis

In the exploratory analysis, we were interested in whether there is a relationship between our six main categories (level 1, but for content reasons, C3 “general evaluation of the situation” was also analyzed on level 2) and demographic variables (age, sex, years of education), depression, perceived stress, resilience, loneliness, health-related quality of life and physical inactivity. Resilience was only recorded from the 2nd corona questionnaire (Coro-Q2) onwards. For this reason, no correlations with our main categories are available for the first corona questionnaire (Coro-Q1).

Results are shown for the six main categories for each of the six questionnaire rounds in Supplementary Tables S3–S9. Although most of the correlations were weak (*r* < 0.3), correlations *r* > 0.1 or correlations that showed a pattern over time were reported. Most of the significant correlations were as expected: Rating the situation as *irrelevant* (C3.2) correlated negatively with fear of COVID-19 and perceived stress, while positive correlations were found with resilience (Coro-Q4/Coro-Q5) and health-related quality of life (Coro-Q4). In contrast, if the situation was rated as stressful (C3.3), a positive correlation with perceived stress and a negative correlation with health-related quality of life emerged as an almost continuous pattern. In addition, a weak negative correlation between rating the situation as stressful and resilience was found in the last three questionnaire rounds. Not surprisingly, at several time points, depression and loneliness also correlated positively with the evaluation of the situation as stressful. For problem-focused strategies (C4), which include (leisure) activities and among them sports, a negative correlation with physical inactivity was found as a consistent pattern. At four time points, fear of COVID-19 also correlated positively with the problem-focused strategies, which include pandemic-related activities (e.g., adhering to Corona rules, seeking information). Emotion-focused strategies (C5), which include maintaining social contacts, showed a negative correlation with loneliness in the last two questionnaire rounds, a pattern of negative correlation with age, and a positive correlation with education in the first two questionnaire rounds. Furthermore, it is worth mentioning a negative correlation of general beliefs (C1) with fear of COVID-19 at two time points (Coro-Q2 and Coro-Q4) and a positive correlation of problem-focused strategies (C4) with years of education (Coro-Q2, Coro-Q3). For total number of codes, there was an almost consistent pattern of a positive correlation with fear of COVID-19 and a negative correlation with physical inactivity. There was also a negative correlation between the total number of codes and age and a positive correlation with years of education in the first two rounds of questionnaires. In the last questionnaire rounds, the total number of codes correlated negatively with loneliness and positively with health-related quality of life. No pattern or noteworthy individual correlations were found for general living conditions (C2), evaluation of the situation as positive (C3.1), and cognitive strategies (C6).

Since gender differences are found in many questionnaires on stress management, resilience, depression, anxiety, and physical activity (Herrera-Añazco et al., 2022; Peyer et al., 2022), we were also exploratively interested in whether these differences could also be found in our categories generated by the qualitative analysis. Regarding gender-related group comparisons using Mann–Whitney *U*-Test, women reported more positive aspects when evaluating the situation at the beginning of the pandemic compared to men (Coro-Q1, Coro-Q2). They also reported more strategies overall in all questionnaire rounds, but especially emotion-focused strategies, showing small effect sizes (*r* between 0.16 and 0.28). In addition, women also reported more problem-focused strategies at four time points. For an overall overview of all correlations and group comparisons, see Supplementary Tables S3–S9.

## 4. Discussion and implications

In the current article, we were interested in how older adults with increased vulnerability for severe COVID-19 cope with the pandemic situation in the long-term. In order to better classify older adults’ coping strategies, a qualitative approach was chosen to identify long-term coping strategies by using a qualitative content analyses according to Mayring (2000, 2015). Contrary to the expectations that older adults might have difficulties withstanding the pandemic situation (Ayalon et al., 2020; Minahan et al., 2021), especially with regard to the psychosocial effects, the results of this article highlight older adults’ resilience in terms of their coping and adaptability during the crisis of COVID-19. Our main finding in this study was that the Lazarus and Folkman (1984) “extended” transactional stress model helped to classify the responses from over 800 participants over a period of 2.5 years.

According to text material, we identified six main categories that comprised the coping strategies mentioned by participants. Categories included three types of coping mechanisms (problem-focused, emotion-focused, or cognitive), as well as general beliefs, living conditions, and the specific evaluation of the situation as positive, irrelevant, or stressful. In line with other studies investigating coping strategies in older adults, the transactional stress model allowed a comprehensive and individual-centered view of the stress-inducing events, such as the pandemic (Minahan et al., 2021; Whitehead and Torossian, 2021). In contrast, the main criticism in this model was the individual-centered view of stress-induced events, without sufficiently considering the situation (e.g., Broda, 1990). The model assumed that an individual experiences stress when he or she perceived an imbalance between him- or herself and the environment, and that this imbalance was classified as a threat.

Especially, for the evaluation of the situation as *irrelevant*, we found that the lower the fear of COVID-19 and the lower the perceived stress, the more text fragments belonging to this category could be coded. We also found a positive correlation between the evaluation of the situation as irrelevant and resilience, which indicated that more resilient individuals were better able to cope with stress and assess situations as stressful less frequently. As expected, the higher the perceived stress and depression and the lower the resilience and health-related quality of life, the more frequently codable text question segments were found to evaluate the situation as *stressful*. In line with our expectations, we found for the problem-focused strategies (C4) including daily structuring, (leisure) activities and sports, that the higher the number in this category, the higher the level of physical activity reported in the standardized questionnaire. Similarly, the correlation with fear of COVID-19 could be explained by the fact that C4 also included a subcategory on “active engagement with the corona pandemic” (e.g., seeking information, following corona rules, taking protective measures, keeping distance). For the emotion-focused strategies, which included the large subcategory of “maintaining and seeking out social contacts,” while it was not surprising that loneliness was negatively correlated with the number of codes in this category, it was interesting that the greater the fear of COVID-19 reported, the higher the number of codes in this category. It probably played a large role that contacts could be maintained at a distance that did not carry a risk of infection, e.g., telephone calls (top 2 among all categories), video conferencing, and messengers. A potential explanation for choosing telephone calls, video conferencing, and messengers might be the rise of the internet and social media platforms (34% of older adults use social media platforms, Anderson and Perrin, 2017). Despite the very weak correlations the pattern of fewer emotion-focused strategies being mentioned with increasing age may be because older adults less frequently use modern means of communication or have smaller social networks. In contrast, there seemed to be a correlation of education with emotion-focused strategies, which might also be explained by the fact that education allowed more opportunities to use different means of communication. The fact that the greater the fear of COVID-19, the higher the total number of codes, was probably since people who were less engaged with the pandemic due to low fear also have a lower need to communicate on this topic (in this case, the question of what helped them deal with the pandemic). The quantitative analysis of the data did confirm several (plausible) relationships between coping and psychosocial factors, which support the validity of our qualitative category system. In other studies, gender differences were found in many questionnaires on stress management, resilience, depression, anxiety, and physical activity, in the sense that women reported be more stressed, more depressed and anxious, and were less physically active (Herrera-Añazco et al., 2022; Peyer et al., 2022); this could be confirmed by our data. Regarding the data on coping during the pandemic, we found that women assessed the situation more positively than men at the beginning of the pandemic (early summer and late fall 2020). It is possible that mostly women responded who had suffered little from the effects of the pandemic and were therefore happy to answer this open-ended question. This finding could possibly also be explained by the fact that in our study women wrote more text overall and achieved a higher number of categories than men. It is already known from other studies that women have a higher need to communicate in open response formats (Moreno and Mayer, 1999). Otherwise, the coping strategies mentioned are consistent with other studies (Finlay et al., 2021; Greenwood-Hickman et al., 2021). For instance, Finlay et al. (2021) reported strategies such as exercising, modifying routines, going outdoors, following public health guidelines, staying socially connected. Negative coping strategies such as overeating were rarely mentioned.

There are several strengths of this study, including (a) a large number of qualitative data collected over 2-year period from over 800 subjects, (b) these data belong to a long-term prospective data collection long before the COVID-19 pandemic in a well-characterized cohort of older adults; (c) continuous rounds of questionnaires with specific questions on pandemic-, health- and psychosocial factors, and (d) an open-ended question about individual coping strategies. The question was deliberately chosen in an open-ended format to allow us to capture the unpredictable developments of the pandemic and not limit ourselves to coping strategies mentioned in already established coping questionnaires (e.g., COPE inventory). However, there are also some limitations that should be mentioned: First, there might have been a bias due to the specific wording of the open question about coping strategies, since examples were given in addition to the specific question (e.g., making phone calls, going for a walk, etc.). This might have led participants to think more about problem-solving strategies and therefore these were mentioned more often in our study. Moreover, the wording of the question about coping strategies seemed to suggest to participants that only positive strategies should be mentioned, so dysfunctional strategies for dealing with the pandemic were only mentioned 1–2 times in all questionnaire rounds. However, the aim was to look at the helpful strategies and not at the obstacles.

Another limitation of our study are missing answers to the question on coping strategies. The question may have been intentionally left unanswered or inadvertently overlooked, or that no coping strategies could be mentioned because nothing was experienced as helpful. Another possible explanation for this finding could be that the participants became tired of answering the question over the duration of the pandemic, in the sense of a lack of motivation. Besides, it should be mentioned that the sample of the TREND study might be selective with respect to well-educated and wealthy individuals. For example, many of our participants reported having their own garden or house, which provided them with free space during the pandemic. But the years of education did only show correlations with coping strategies lower than 0.07 (*cf.* Supplementary Tables S2–S7).

Methodologically, it should be noted that the coping strategies were recorded by means of a free-text field and using an open format question, rather than using an already established coping questionnaire. Nevertheless, the results of these surveys are unique, as data on coping strategies were collected at regular intervals over a period of 2.5 years, which extend the data pool of usual qualitative surveys (in our study, a total of 20,578 text segments in 4561 records, originating from more than 800 participants and collected at six time points over a 2.5-year period, were coded). The response rates over 2.5 years stayed between 83 and 90% and were exceptionally high for surveys. These constantly high rates prevent a severe bias towards healthy and resilient subjects, which is underlined by the fact that similar patterns of coping strategies emerged, even when all respondents were included, and analyses were not limited to subjects who participated in each of the six questionnaires. Society should be aware of helpful strategies, share them with older individuals and support and facilitate such strategies and activities, as the next pandemic and lockdowns might come.

In conclusion, the present findings provide novel insights into the longitudinal coping strategies of older adults during the COVID-19 pandemic. Throughout the COVID-19 pandemic, emotional-focused as well as problem-focused strategies were the main coping strategies, whereas general beliefs, general living conditions and the evaluation were mentioned less frequently. However, the current results so far do not allow a conclusion on how stable these strategies were for the individual.

## Data availability statement

The original contributions presented in the study are included in the article/Supplementary material, further inquiries can be directed to the corresponding author.

## Ethics statement

The TREND study and all its amendments, including the questionnaires during the COVID-19 pandemic, were approved by the ethics committee at the Medical Faculty of the Eberhard Karls University and at the University Hospital of Tübingen (No. 90/2009BO2). The study was conducted in accordance with the local legislation and institutional requirements. The participants provided their written informed consent to participate in this study.

## Author contributions

SH, DB, WM, KB, US, A-KT, TD, GE, and AT developed the research question, and contributed to the conception and study design. LK, CM, US, A-KT, SH, GE, and AT implemented the questionnaires. US programmed the database and online questionnaires. A-KT, US, and LK entered the data. US, LK, GE, and AT categorized the data in MAXQDA. LK and US contributed to drafting the text, designed the figures and tables, and performed the statistical analyses. All authors provided critical feedback and helped in every stage of the research, analysis, and manuscript. All authors contributed to the article and approved the submitted version.

## Funding

The TREND study is being conducted at the University Hospital Tübingen and has been supported by the Hertie Institute for Clinical Brain Research, the DZNE, the Geriatric Center of Tübingen, the Center for Integrative Neuroscience. and partially funded in subprojects by the Teva Pharmaceutical Industries Union Chimique Belge, Janssen Pharmaceuticals, the International Parkinson Foundation before 2017. Specifically, the CORO-TREND project of the TREND study was funded by the German Research Society (DFG, grant number: AOBJ: 675915, Project 458531848).

## Conflict of interest

The authors declare that the research was conducted in the absence of any commercial or financial relationships that could be construed as a potential conflict of interest.

## Publisher’s note

All claims expressed in this article are solely those of the authors and do not necessarily represent those of their affiliated organizations, or those of the publisher, the editors and the reviewers. Any product that may be evaluated in this article, or claim that may be made by its manufacturer, is not guaranteed or endorsed by the publisher.

## Footnotes

^1^ www.trend-studie.de
