# Supplementary material for: Older adults’ coping strategies during the COVID-19 pandemic – a longitudinal mixed-methods study
Source: Front Psychol. 2023 Sep 4;14:1209021. doi: 10.3389/fpsyg.2023.1209021 (PMC10512280; doi:10.3389/fpsyg.2023.1209021)
Supplement: Supplementary file 1 [file Data_Sheet_1.docx]

| **Table S1**  *Prodromal markers (depression, hyposmia, probable RBD) at first TREND study visit* | | |  |  |  |
| --- | --- | --- | --- | --- | --- |
|  | **all TREND participants** | |  | **only TREND participants who took part in at least one Corona questionnaire** | |
|  | *N* = 1201 | |  | *N* = 880 | |
|  | *n* | % |  | *n* | % |
| HC | 484 | 40% |  | 390 | 44% |
| DEP | 170 | 14% |  | 130 | 15% |
| HYP | 248 | 21% |  | 161 | 18% |
| RBD | 55 | 5% |  | 43 | 5% |
| DEP+HYP | 78 | 6% |  | 52 | 6% |
| DEP+RBD | 64 | 5% |  | 46 | 5% |
| HYP+RBD | 56 | 5% |  | 34 | 4% |
| DEP+HYP+RBD | 46 | 4% |  | 24 | 3% |
| **total** | **1201** | **100%** |  | **880** | **100%** |
| 0 prodromal markers | 484 | 40% |  | 390 | 44% |
| 1 prodromal marker | 473 | 39% |  | 334 | 38% |
| 2 prodromal markers | 198 | 16% |  | 132 | 15% |
| 3 prodromal markers | 46 | 4% |  | 24 | 3% |
| **total** | **1201** | **100%** |  | **880** | **100%** |
| DEP | 358 | 30% |  | 252 | 29% |
| HYP | 428 | 36% |  | 271 | 31% |
| RBD | 221 | 18% |  | 147 | 17% |

*Note.* HC = healthy control (no prodromal markers: no depression, no hyposmia, no probable RBD), DEP = current depressive episode (ICD-10, DSM-IV) or lifetime depression, HYP = hyposmia (Sniffin' Sticks 16 identification ≤11; exclusion of participants with current cold, sinusitis or hay fever symptoms who showed poor test results), RBD = probable REM sleep behavior disorder (REM Sleep Behavior Screening Questionnaire ≥5)

**Table S2**

*Number of mentions for each category for all six questionnaire rounds and in total*

| **Code No.** | **Level 1** | **Level 2** | **Level 3** | **Level 4** | **Level 5** | **Level 6** | **Level 7** | **Coro-Q1** | **Coro-Q2** | **Coro-Q3** | **Coro-Q4** | **Coro-Q5** | **Coro-Q6** | **sum (all questionnaire rounds)** |
| --- | --- | --- | --- | --- | --- | --- | --- | --- | --- | --- | --- | --- | --- | --- |
| 1 | General Beliefs (concepts, values, convictions) | | |  |  |  |  | 0 | 0 | 0 | 0 | 0 | 0 | **0** |
| 2 |  | Life Attitude and Experience | |  |  |  |  | 0 | 1 | 2 | 3 | 3 | 0 | **9** |
| 3 |  |  | Positive Thinking/Optimism | |  |  |  | 11 | 18 | 7 | 6 | 11 | 8 | **61** |
| 4 |  |  | Self-Motivation |  |  |  |  | 0 | 0 | 0 | 0 | 0 | 0 | **0** |
| 5 |  |  |  | Discipline |  |  |  | 1 | 1 | 1 | 0 | 0 | 1 | **4** |
| 6 |  |  |  | (Own) Strong Will |  |  |  | 0 | 0 | 2 | 1 | 1 | 0 | **4** |
| 7 |  |  |  | Self-Initiative |  |  |  | 0 | 0 | 1 | 1 | 0 | 0 | **2** |
| 8 |  |  | Resilience |  |  |  |  | 0 | 3 | 0 | 2 | 0 | 1 | **6** |
| 9 |  |  |  | Radical Acceptance | |  |  | 0 | 3 | 2 | 1 | 0 | 1 | **7** |
| 10 |  |  |  | Self-Confidence |  |  |  | 3 | 1 | 1 | 1 | 1 | 1 | **8** |
| 11 |  |  |  | Reflectiveness |  |  |  | 0 | 0 | 1 | 1 | 0 | 0 | **2** |
| 12 |  |  |  | Solution Orientation | |  |  | 0 | 1 | 1 | 0 | 0 | 1 | **3** |
| 13 |  |  |  | Patience |  |  |  | 0 | 0 | 1 | 3 | 0 | 0 | **4** |
| 14 |  |  | Joy of Life |  |  |  |  | 1 | 0 | 0 | 1 | 1 | 2 | **5** |
| 15 |  |  |  | Creativity |  |  |  | 1 | 3 | 3 | 4 | 0 | 0 | **11** |
| 16 |  |  |  | Humor |  |  |  | 4 | 3 | 3 | 2 | 0 | 2 | **14** |
| 17 |  |  |  | Empathy |  |  |  | 0 | 1 | 0 | 0 | 0 | 0 | **1** |
| 18 |  |  |  | Kindness & Helpfulness | |  |  | 1 | 1 | 0 | 0 | 0 | 0 | **2** |
| 19 |  | Faith/Spirituality |  |  |  |  |  | 7 | 15 | 5 | 3 | 2 | 2 | **34** |
| 20 |  | Own Health Status |  |  |  |  |  | 12 | 9 | 6 | 6 | 4 | 3 | **40** |
| 21 |  | Introverted/used to Being Alone | |  |  |  |  | 4 | 2 | 3 | 3 | 2 | 3 | **17** |
| 22 | General Living Conditions (material/financial/social) | | |  |  |  |  | 0 | 0 | 0 | 0 | 0 | 0 | **0** |
| 23 |  | Good Health System/Medical Care | |  |  |  |  | 3 | 1 | 0 | 0 | 0 | 0 | **4** |
| 24 |  | Not Being/Living Alone | |  |  |  |  | 1 | 2 | 0 | 2 | 1 | 1 | **7** |
| 25 |  | Autonomy/Independence/Self-Reliance | |  |  |  |  | 2 | 3 | 2 | 0 | 1 | 1 | **9** |
| 26 |  | Be Retired |  |  |  |  |  | 12 | 12 | 3 | 4 | 4 | 1 | **36** |
| 27 |  | Financial Security |  |  |  |  |  | 12 | 12 | 1 | 6 | 1 | 4 | **36** |
| 28 |  | Housing Situation |  |  |  |  |  | 2 | 2 | 4 | 1 | 8 | 1 | **18** |
| 29 |  |  | Good Living Environment | |  |  |  | 2 | 2 | 1 | 3 | 0 | 3 | **11** |
| 30 |  |  |  | Infrastructure (Stores, Supermarket etc. available) | | |  | 1 | 3 | 3 | 0 | 1 | 0 | **8** |
| 31 |  |  |  | Living in the Countryside/a Village | |  |  | 15 | 5 | 4 | 4 | 1 | 3 | **32** |
| 32 |  |  |  | Living Close to the Countryside/Outskirts of Town | | |  | 5 | 0 | 2 | 0 | 0 | 3 | **10** |
| 33 |  |  | Living Atmosphere (nice apartment, feeling comfortable at home) | | | |  | 6 | 3 | 4 | 8 | 2 | 3 | **26** |
| 34 |  |  | Specific Housing Situation/Amenities | |  |  |  | 0 | 0 | 0 | 0 | 0 | 1 | **1** |
| 35 |  |  |  | Enough Living Space/Lots of Living Space | | |  | 8 | 4 | 4 | 1 | 3 | 3 | **23** |
| 36 |  |  |  | Balcony/Terrace |  |  |  | 16 | 13 | 5 | 3 | 0 | 2 | **39** |
| 37 |  |  |  | Garden |  |  |  | 180 | 166 | 167 | 82 | 95 | 76 | **766** |
| 38 |  |  |  | Plot of Land |  |  |  | 3 | 0 | 1 | 1 | 1 | 3 | **9** |
| 39 |  |  |  | Own House/ living in a House | |  |  | 47 | 10 | 24 | 12 | 6 | 7 | **106** |
| 40 |  |  |  | Own Apartment/ living in an Apartment | | |  | 4 | 0 | 0 | 1 | 0 | 0 | **5** |
| 41 |  | Mobility |  |  |  |  |  | 1 | 2 | 1 | 3 | 0 | 1 | **8** |
| 42 |  |  | Bicycling/Walking instead of Public Transport | | |  |  | 1 | 0 | 4 | 2 | 2 | 1 | **10** |
| 43 |  |  | Mobility by Public Transport | |  |  |  | 0 | 2 | 3 | 1 | 2 | 2 | **10** |
| 44 |  |  | Mobility by own Means of Transport | |  |  |  | 0 | 0 | 1 | 0 | 0 | 0 | **1** |
| 45 |  |  |  | Camper |  |  |  | 0 | 6 | 1 | 12 | 4 | 6 | **29** |
| 46 |  |  |  | Own Car |  |  |  | 5 | 15 | 6 | 10 | 5 | 6 | **47** |
| 47 |  |  |  | Company Car |  |  |  | 1 | 0 | 0 | 0 | 0 | 0 | **1** |
| 48 | General Evaluation of the Situation (Meta-Reflection) | | |  |  |  |  | 0 | 0 | 0 | 0 | 0 | 0 | **0** |
| 49 |  | Positive |  |  |  |  |  | 4 | 2 | 1 | 0 | 0 | 0 | **7** |
| 50 |  |  | Better/Longer Sleep (Quality) | |  |  |  | 3 | 1 | 1 | 0 | 0 | 0 | **5** |
| 51 |  |  | Having More Time |  |  |  |  | 17 | 7 | 3 | 0 | 2 | 1 | **30** |
| 52 |  |  |  | Less Hectic; More Serenity/Calmness | |  |  | 22 | 6 | 1 | 1 | 0 | 2 | **32** |
| 53 |  |  |  |  | Less Noise |  |  | 2 | 0 | 0 | 0 | 0 | 0 | **2** |
| 54 |  |  |  | Less to do |  |  |  | 0 | 3 | 0 | 0 | 0 | 0 | **3** |
| 55 |  |  |  | Free Time Management | |  |  | 1 | 0 | 0 | 1 | 0 | 0 | **2** |
| 56 |  |  | No Boredom |  |  |  |  | 2 | 7 | 2 | 4 | 5 | 4 | **24** |
| 57 |  |  | Freedom from Obligations/Appointments | | |  |  | 12 | 10 | 3 | 1 | 0 | 0 | **26** |
| 58 |  |  | Less People outside | |  |  |  | 3 | 2 | 0 | 0 | 0 | 0 | **5** |
| 59 |  |  | (Intensive) Connection with Others | |  |  |  | 2 | 0 | 3 | 0 | 0 | 1 | **6** |
| 60 |  |  |  | Distance to People | |  |  | 1 | 2 | 1 | 0 | 1 | 1 | **6** |
| 61 |  | Irrelevant |  |  |  |  |  | 0 | 0 | 0 | 0 | 0 | 0 | **0** |
| 62 |  |  | Pandemic not as Threatening as Before | |  |  |  | 0 | 0 | 0 | 0 | 0 | 1 | **1** |
| 63 |  |  | There were more important Things than the Pandemic | | |  |  | 0 | 4 | 0 | 2 | 1 | 6 | **13** |
| 64 |  |  | No Restriction/Change due to Pandemic | | |  |  | 20 | 18 | 9 | 8 | 15 | 25 | **95** |
| 65 |  |  |  | Lived on Normally |  |  |  | 28 | 16 | 3 | 7 | 23 | 18 | **95** |
| 66 |  |  |  | Life goes on |  |  |  | 0 | 1 | 1 | 0 | 0 | 0 | **2** |
| 67 |  |  |  | Nothing (special) |  |  |  | 1 | 1 | 5 | 0 | 3 | 3 | **13** |
| 68 |  |  | Hardly any Restriction/Change due to Pandemic | | |  |  | 20 | 18 | 8 | 8 | 16 | 14 | **84** |
| 69 |  |  | No special Support needed during the Pandemic | | |  |  | 1 | 1 | 0 | 3 | 2 | 1 | **8** |
| 70 |  |  | Do not miss Personal Contacts | |  |  |  | 1 | 0 | 1 | 0 | 0 | 0 | **2** |
| 71 |  |  | No Fear (of Corona/Infection) | |  |  |  | 2 | 0 | 1 | 3 | 3 | 2 | **11** |
| 72 |  |  | Joy/Relief about Relaxation of Corona Rules | | |  |  | 7 | 1 | 0 | 1 | 4 | 2 | **15** |
| 73 |  | Stressful |  |  |  |  |  | 0 | 0 | 0 | 2 | 0 | 0 | **2** |
| 74 |  |  | Damage/Loss |  |  |  |  | 0 | 0 | 1 | 0 | 0 | 0 | **1** |
| 75 |  |  |  | (Severe) Illness/Surgery during Pandemic | | |  | 6 | 9 | 5 | 6 | 2 | 7 | **35** |
| 76 |  |  |  |  | Depression |  |  | 0 | 0 | 0 | 0 | 0 | 1 | **1** |
| 77 |  |  |  | Weight Gain |  |  |  | 0 | 1 | 1 | 0 | 0 | 0 | **2** |
| 78 |  |  |  | Restrictions on Medical Care | |  |  | 0 | 0 | 0 | 0 | 0 | 0 | **0** |
| 79 |  |  |  |  | Doctor's Appointments difficult to get | |  | 0 | 0 | 0 | 1 | 0 | 0 | **1** |
| 80 |  |  |  |  | Medical Treatment postponed | |  | 2 | 0 | 0 | 0 | 0 | 0 | **2** |
| 81 |  |  |  | Loss of Social Contacts | |  |  | 13 | 8 | 8 | 5 | 6 | 3 | **43** |
| 82 |  |  |  |  | No Physical Contact/Hugs | |  | 2 | 0 | 1 | 0 | 0 | 0 | **3** |
| 83 |  |  |  | Cancellation of Events | |  |  | 3 | 2 | 4 | 3 | 5 | 1 | **18** |
| 84 |  |  |  | Travel Restrictions |  |  |  | 9 | 9 | 3 | 1 | 3 | 1 | **26** |
| 85 |  |  |  | Restaurants closed | |  |  | 3 | 2 | 2 | 0 | 1 | 0 | **8** |
| 86 |  |  |  | Restrictions on Sports Activities | |  |  | 8 | 7 | 6 | 1 | 1 | 0 | **23** |
| 87 |  |  |  | Loss of Hobbies |  |  |  | 4 | 1 | 2 | 2 | 2 | 0 | **11** |
| 88 |  |  |  | Loss of Volunteer Work | |  |  | 1 | 2 | 0 | 0 | 1 | 0 | **4** |
| 89 |  |  |  | Short-time Work |  |  |  | 1 | 1 | 0 | 0 | 0 | 0 | **2** |
| 90 |  |  |  | Nothing helped |  |  |  | 1 | 1 | 4 | 1 | 2 | 2 | **11** |
| 91 |  |  | Threat |  |  |  |  | 0 | 1 | 1 | 2 | 0 | 0 | **4** |
| 92 |  |  |  | Others do not Follow Rules/are Inconsiderate | | |  | 0 | 1 | 0 | 0 | 0 | 0 | **1** |
| 93 |  |  |  | Worry about getting COVID yourself | |  |  | 0 | 1 | 0 | 1 | 0 | 1 | **3** |
| 94 |  |  |  | Refusal of the Corona Vaccination | |  |  | 1 | 0 | 1 | 2 | 1 | 1 | **6** |
| 95 |  |  |  | Politics & their Decisions | |  |  | 6 | 3 | 3 | 7 | 2 | 1 | **22** |
| 96 |  |  |  | Bad Course of the Pandemic | |  |  | 0 | 0 | 0 | 0 | 2 | 0 | **2** |
| 97 |  |  |  | Worry/Burden because of the War and its Consequences | | |  | 0 | 0 | 0 | 0 | 10 | 0 | **10** |
| 98 |  |  |  | Interpersonal Conflicts | |  |  | 1 | 0 | 2 | 0 | 0 | 0 | **3** |
| 99 |  |  |  | Worry about getting Dementia/Memory Problems | | |  | 0 | 0 | 0 | 0 | 0 | 2 | **2** |
| 100 |  |  |  | (Imminent) Homelessness | |  |  | 0 | 0 | 0 | 0 | 1 | 1 | **2** |
| 101 |  |  | Challenge |  |  |  |  | 0 | 1 | 0 | 0 | 0 | 0 | **1** |
| 102 |  |  |  | Not Meeting own Standards | |  |  | 0 | 0 | 0 | 0 | 1 | 0 | **1** |
| 103 |  |  |  | Too little Energy/Exhaustion | |  |  | 0 | 0 | 1 | 2 | 0 | 0 | **3** |
| 104 |  |  |  | Have too Little Time | |  |  | 0 | 1 | 0 | 3 | 2 | 0 | **6** |
| 105 |  |  |  | Coping with a Move | |  |  | 2 | 2 | 2 | 0 | 4 | 2 | **12** |
| 106 |  |  |  | (Serious) Illness/Death of a close Person | | |  | 2 | 5 | 2 | 3 | 5 | 1 | **18** |
| 107 |  |  |  | Restrictions Nursing Home/Care Facility | | |  | 1 | 0 | 1 | 1 | 1 | 1 | **5** |
| 108 |  |  |  | Health Problems |  |  |  | 11 | 11 | 13 | 7 | 8 | 3 | **53** |
| 109 |  |  |  | Too few Vaccination Possibilities | |  |  | 0 | 0 | 2 | 2 | 0 | 0 | **4** |
| 110 |  |  |  | Shopping more Complicated | |  |  | 0 | 0 | 2 | 0 | 0 | 0 | **2** |
| 111 |  |  |  | Instrumental Support (e.g. cleaning help) difficult to get | | |  | 0 | 0 | 0 | 1 | 0 | 0 | **1** |
| 112 |  |  |  | Difficulties in Dealing with Corona Rules | | |  | 0 | 0 | 0 | 0 | 0 | 1 | **1** |
| 113 |  |  |  |  | Suffering from Restrictions | |  | 0 | 3 | 0 | 0 | 0 | 0 | **3** |
| 114 |  |  |  |  | Suffering from Isolation/Lockdown | |  | 3 | 0 | 0 | 0 | 0 | 0 | **3** |
| 115 |  |  |  |  | Difficulties with Masks | |  | 2 | 3 | 1 | 0 | 1 | 4 | **11** |
| 116 |  |  |  |  | Difficulties with Curfew | |  | 2 | 0 | 0 | 0 | 0 | 0 | **2** |
| 117 |  |  |  |  | Difficulties with Hygiene Rules | |  | 1 | 0 | 0 | 1 | 0 | 0 | **2** |
| 118 | Problem-focused Strategies | |  |  |  |  |  | 0 | 0 | 0 | 0 | 0 | 0 | **0** |
| 119 |  | Active Engagement with the Corona Pandemic | | |  |  |  | 0 | 0 | 0 | 0 | 0 | 0 | **0** |
| 120 |  |  | Information Gathering | |  |  |  | 45 | 19 | 14 | 8 | 4 | 5 | **95** |
| 121 |  |  | Compliant Behavior | | |  |  | 0 | 2 | 0 | 0 | 0 | 1 | **3** |
| 122 |  |  |  | Using testing Opportunities (Corona) | |  |  | 0 | 0 | 4 | 0 | 1 | 4 | **9** |
| 123 |  |  |  | Getting Vaccination against Corona | |  |  | 0 | 0 | 14 | 18 | 8 | 16 | **56** |
| 124 |  |  |  | Adhere to Corona Rules | |  |  | 21 | 19 | 9 | 11 | 11 | 7 | **78** |
| 125 |  |  |  |  | Reduce Social Contact | |  | 15 | 12 | 6 | 3 | 5 | 3 | **44** |
| 126 |  |  |  |  |  | Less Physical Contact (no handshake, greeting kiss) | | 0 | 0 | 0 | 0 | 0 | 2 | **2** |
| 127 |  |  |  |  |  | Stay at Home |  | 5 | 6 | 2 | 2 | 3 | 1 | **19** |
| 128 |  |  |  |  |  | Avoid Crowds |  | 5 | 5 | 1 | 3 | 1 | 7 | **22** |
| 129 |  |  |  |  |  | Avoid Public Transportation | | 3 | 4 | 0 | 1 | 0 | 0 | **8** |
| 130 |  |  |  |  |  | Less/No Shopping |  | 28 | 2 | 2 | 1 | 0 | 0 | **33** |
| 131 |  |  |  |  |  | Few/No Visits to the Doctor | | 4 | 0 | 0 | 1 | 0 | 0 | **5** |
| 132 |  |  |  |  |  | Cancel Trips/Events | | 2 | 2 | 1 | 3 | 1 | 5 | **14** |
| 133 |  |  |  |  |  | Work from Remote | | 4 | 5 | 1 | 1 | 0 | 0 | **11** |
| 134 |  |  |  |  | Visits/Meetings/Contacts that Comply with Corona Rules | | | 49 | 38 | 30 | 24 | 8 | 13 | **162** |
| 135 |  |  |  |  | Wear Masks |  |  | 5 | 16 | 2 | 2 | 5 | 20 | **50** |
| 136 |  |  |  |  | Keep Distance |  |  | 4 | 19 | 3 | 2 | 3 | 4 | **35** |
| 137 |  |  |  |  | Disinfection/Hygiene | |  | 0 | 5 | 0 | 1 | 1 | 1 | **8** |
| 138 |  |  | Caution and Consideration | |  |  |  | 0 | 0 | 0 | 0 | 0 | 0 | **0** |
| 139 |  |  |  | Mutual Consideration | |  |  | 1 | 2 | 0 | 0 | 0 | 1 | **4** |
| 140 |  |  |  | Careful Behavior |  |  |  | 5 | 7 | 3 | 1 | 3 | 8 | **27** |
| 141 |  |  | Talking to Others about Corona | |  |  |  | 5 | 3 | 2 | 0 | 5 | 3 | **18** |
| 142 |  |  | Critical Questioning of Corona | |  |  |  | 2 | 3 | 3 | 5 | 0 | 3 | **16** |
| 143 |  |  | Trust in Government/Politics/Authorities/Measures | | |  |  | 13 | 3 | 2 | 0 | 1 | 0 | **19** |
| 144 |  |  | Active Criticism of and Resistance to Corona Rules | | |  |  | 0 | 0 | 0 | 0 | 0 | 0 | **0** |
| 145 |  |  |  | Community with like-minded People (Corona/Vaccine critical) | | | | 0 | 0 | 1 | 1 | 0 | 1 | **3** |
| 146 |  |  |  | Attend demonstrations | |  |  | 1 | 1 | 0 | 0 | 1 | 0 | **3** |
| 147 |  |  |  | Disobey Corona Rules | |  |  | 1 | 1 | 2 | 1 | 1 | 0 | **6** |
| 148 |  |  | Knowledge about Health Status of Relatives | | |  |  | 8 | 4 | 1 | 2 | 1 | 2 | **18** |
| 149 |  |  | No/Less Preoccupation with Pandemic | |  |  |  | 3 | 4 | 2 | 1 | 4 | 2 | **16** |
| 150 |  | Structuring Everyday Life | |  |  |  |  | 11 | 5 | 3 | 3 | 8 | 5 | **35** |
| 151 |  |  | Everyday Tasks |  |  |  |  | 10 | 5 | 6 | 4 | 6 | 4 | **35** |
| 152 |  |  |  | Housework/Household | |  |  | 38 | 29 | 36 | 29 | 23 | 12 | **167** |
| 153 |  |  |  |  | Cleaning |  |  | 5 | 1 | 1 | 3 | 2 | 0 | **12** |
| 154 |  |  |  | Prepare Meals |  |  |  | 1 | 1 | 0 | 0 | 0 | 0 | **2** |
| 155 |  |  |  |  | Try new Recipes/Dishes | |  | 6 | 4 | 5 | 1 | 1 | 4 | **21** |
| 156 |  |  |  |  | Barbecue |  |  | 1 | 2 | 0 | 0 | 0 | 0 | **3** |
| 157 |  |  |  |  | Cooking |  |  | 33 | 27 | 29 | 21 | 12 | 18 | **140** |
| 158 |  |  |  |  | Baking |  |  | 8 | 11 | 6 | 6 | 4 | 4 | **39** |
| 159 |  |  |  | Gardening |  |  |  | 129 | 124 | 126 | 65 | 69 | 64 | **577** |
| 160 |  |  |  | Use Health System | |  |  | 0 | 0 | 0 | 0 | 0 | 0 | **0** |
| 161 |  |  |  |  | Supply of Medicines | |  | 3 | 0 | 1 | 0 | 0 | 0 | **4** |
| 162 |  |  |  |  | Seeing a Doctor |  |  | 4 | 5 | 5 | 5 | 2 | 2 | **23** |
| 163 |  |  |  |  | Psychotherapy/Psychiatric Care | |  | 2 | 1 | 1 | 1 | 3 | 2 | **10** |
| 164 |  |  |  |  | Physical Therapy |  |  | 2 | 2 | 3 | 2 | 4 | 1 | **14** |
| 165 |  |  |  |  | Therapy (type unknown) | |  | 1 | 0 | 0 | 1 | 0 | 0 | **2** |
| 166 |  |  |  |  | Rehabilitation |  |  | 3 | 4 | 2 | 2 | 2 | 3 | **16** |
| 167 |  |  |  | Renovate/DIY Home improvement/Remodeling | | |  | 25 | 24 | 25 | 12 | 10 | 15 | **111** |
| 168 |  |  |  |  | Declutter |  |  | 11 | 6 | 8 | 1 | 0 | 4 | **30** |
| 169 |  |  |  |  | Clean up |  |  | 25 | 7 | 8 | 0 | 2 | 8 | **50** |
| 170 |  |  |  | Bureaucracy (e.g. Tax Return) | |  |  | 5 | 5 | 5 | 0 | 2 | 6 | **23** |
| 171 |  |  |  | Tasks |  |  |  | 4 | 0 | 0 | 0 | 0 | 0 | **4** |
| 172 |  |  |  |  | (Grocery) Shopping | |  | 37 | 25 | 42 | 24 | 10 | 13 | **151** |
| 173 |  |  |  |  | Ensure the Supply of Food | |  | 1 | 0 | 0 | 0 | 0 | 0 | **1** |
| 174 |  |  |  |  |  | Have Food Supplies | | 1 | 0 | 0 | 0 | 0 | 0 | **1** |
| 175 |  |  |  |  |  | Takeaway Food |  | 0 | 3 | 5 | 0 | 0 | 0 | **8** |
| 176 |  |  |  |  | Visit to the Hairdresser | |  | 0 | 1 | 1 | 0 | 0 | 0 | **2** |
| 177 |  |  |  |  | Use a Delivery Service | |  | 5 | 2 | 1 | 1 | 0 | 0 | **9** |
| 178 |  |  |  |  | Use Online Stores and Online Banking | |  | 2 | 4 | 2 | 0 | 0 | 0 | **8** |
| 179 |  |  |  | Occupation/Work |  |  |  | 41 | 44 | 43 | 43 | 30 | 29 | **230** |
| 180 |  |  |  |  | Teaching |  |  | 0 | 1 | 0 | 2 | 1 | 1 | **5** |
| 181 |  |  |  | Volunteer Work |  |  |  | 9 | 17 | 26 | 20 | 20 | 27 | **119** |
| 182 |  |  |  | Help/Support Others, Care for Others | |  |  | 1 | 5 | 2 | 1 | 0 | 3 | **12** |
| 183 |  |  |  |  | Help/Support/Care (for) Family Members | | | 2 | 2 | 2 | 1 | 2 | 6 | **15** |
| 184 |  |  |  |  |  | Care for/Support Spouse | | 1 | 9 | 3 | 3 | 4 | 8 | **28** |
| 185 |  |  |  |  |  | Care for/Support Siblings | | 1 | 0 | 1 | 0 | 1 | 0 | **3** |
| 186 |  |  |  |  |  | Care for/Support own Children | | 1 | 2 | 1 | 2 | 1 | 1 | **8** |
| 187 |  |  |  |  |  | Care for/Support Parents | | 2 | 5 | 4 | 3 | 1 | 2 | **17** |
| 188 |  |  |  |  |  | Care for Grandchildren | | 5 | 16 | 10 | 21 | 10 | 11 | **73** |
| 189 |  |  |  |  | Help/Support/Care (for) Others (not family) | | | 2 | 0 | 2 | 0 | 1 | 1 | **6** |
| 190 |  |  |  |  |  | Help/Support Friends | | 1 | 2 | 2 | 1 | 2 | 0 | **8** |
| 191 |  |  |  |  |  | Help neighbors |  | 1 | 2 | 0 | 2 | 1 | 1 | **7** |
| 192 |  |  |  |  |  | Help Refugees |  | 0 | 0 | 1 | 1 | 0 | 2 | **4** |
| 193 |  |  |  |  |  | Care for Children |  | 0 | 1 | 0 | 1 | 1 | 3 | **6** |
| 194 |  |  |  | Work on Things that have been left undone | | |  | 10 | 7 | 0 | 0 | 0 | 0 | **17** |
| 195 |  |  | (Leisure) Activities |  |  |  |  | 19 | 3 | 5 | 3 | 5 | 10 | **45** |
| 196 |  |  |  | Hobbies |  |  |  | 5 | 4 | 14 | 9 | 11 | 6 | **49** |
| 197 |  |  |  |  | Hobby in a Group |  |  | 0 | 0 | 1 | 1 | 2 | 1 | **5** |
| 198 |  |  |  |  | Pursue Interests |  |  | 2 | 5 | 2 | 3 | 0 | 2 | **14** |
| 199 |  |  |  |  |  | Research (not Corona related) | | 0 | 0 | 3 | 1 | 0 | 2 | **6** |
| 200 |  |  |  |  |  | Scientific Work (outside of Professional Activities) | | 0 | 1 | 0 | 0 | 3 | 1 | **5** |
| 201 |  |  |  |  |  | Interest in News/Daily Events | | 19 | 7 | 27 | 9 | 11 | 17 | **90** |
| 202 |  |  |  |  |  | Art/Artistic Topics |  | 4 | 3 | 1 | 1 | 3 | 1 | **13** |
| 203 |  |  |  |  |  | Natural Sciences & Mathematics | | 0 | 0 | 0 | 0 | 0 | 0 | **0** |
| 204 |  |  |  |  |  |  | Mathematics | 1 | 1 | 1 | 1 | 0 | 0 | **4** |
| 205 |  |  |  |  |  |  | Animals | 0 | 0 | 1 | 0 | 0 | 0 | **1** |
| 206 |  |  |  |  |  |  | Environmental Protection & Climate Change | 1 | 1 | 0 | 0 | 0 | 0 | **2** |
| 207 |  |  |  |  |  |  | Ornithology | 1 | 0 | 1 | 0 | 0 | 0 | **2** |
| 208 |  |  |  |  |  | Technology |  | 0 | 0 | 0 | 0 | 0 | 0 | **0** |
| 209 |  |  |  |  |  |  | Vintage Cars | 1 | 0 | 0 | 0 | 0 | 0 | **1** |
| 210 |  |  |  |  |  |  | Programming | 1 | 0 | 1 | 1 | 0 | 0 | **3** |
| 211 |  |  |  |  |  |  | Amateur Radio | 0 | 1 | 1 | 0 | 0 | 0 | **2** |
| 212 |  |  |  |  |  |  | Electronics | 0 | 1 | 1 | 0 | 0 | 0 | **2** |
| 213 |  |  |  |  |  | Politics & History |  | 1 | 0 | 0 | 0 | 0 | 0 | **1** |
| 214 |  |  |  |  |  |  | Politics | 1 | 3 | 0 | 4 | 0 | 3 | **11** |
| 215 |  |  |  |  |  |  | World War 2 | 1 | 0 | 0 | 0 | 0 | 0 | **1** |
| 216 |  |  |  |  |  | Psychology & Sociology | | 0 | 1 | 0 | 0 | 0 | 0 | **1** |
| 217 |  |  |  |  |  | Medicine |  | 1 | 0 | 0 | 0 | 0 | 1 | **2** |
| 218 |  |  |  |  |  | Philosophy |  | 0 | 1 | 0 | 0 | 0 | 1 | **2** |
| 219 |  |  |  |  |  | Genealogy |  | 2 | 0 | 1 | 0 | 0 | 0 | **3** |
| 220 |  |  |  |  |  | Other Intellectual Interests & Activities | | 1 | 3 | 2 | 0 | 1 | 1 | **8** |
| 221 |  |  |  |  | Reading |  |  | 116 | 95 | 124 | 86 | 96 | 82 | **599** |
| 222 |  |  |  |  | Writing |  |  | 8 | 6 | 4 | 5 | 2 | 3 | **28** |
| 223 |  |  |  |  | Learning foreign Languages/Attending Language Courses | | | 2 | 3 | 6 | 7 | 13 | 5 | **36** |
| 224 |  |  |  |  | Handicrafts (e.g. Knitting, Sewing) | |  | 26 | 19 | 28 | 19 | 17 | 13 | **122** |
| 225 |  |  |  |  | Fine Arts/Arts and Crafts | |  | 0 | 0 | 0 | 0 | 0 | 3 | **3** |
| 226 |  |  |  |  |  | Arts and Crafts/Handicrafts as a Hobby | | 5 | 4 | 4 | 2 | 3 | 3 | **21** |
| 227 |  |  |  |  |  | Painting/Drawing |  | 4 | 6 | 9 | 2 | 4 | 3 | **28** |
| 228 |  |  |  |  |  | Calligraphy |  | 1 | 0 | 0 | 1 | 1 | 0 | **3** |
| 229 |  |  |  |  | Photography (Taking Photos, Viewing/Editing Photos, etc.) | | | 9 | 6 | 6 | 1 | 4 | 0 | **26** |
| 230 |  |  |  |  | Technical Hobby |  |  | 0 | 0 | 0 | 0 | 0 | 0 | **0** |
| 231 |  |  |  |  |  | Model Railroad |  | 1 | 1 | 0 | 0 | 0 | 1 | **3** |
| 232 |  |  |  |  |  | Restoring/Maintaining/Repairing Vehicles | | 1 | 2 | 3 | 2 | 1 | 0 | **9** |
| 233 |  |  |  |  | Making Music (Playing Instruments/Singing/Composing) | | | 17 | 20 | 17 | 17 | 18 | 25 | **114** |
| 234 |  |  |  |  | Listening |  |  | 0 | 0 | 0 | 0 | 0 | 0 | **0** |
| 235 |  |  |  |  |  | Music |  | 16 | 23 | 14 | 17 | 12 | 11 | **93** |
| 236 |  |  |  |  |  | Radio |  | 15 | 15 | 11 | 8 | 4 | 7 | **60** |
| 237 |  |  |  |  |  | Podcasts |  | 4 | 1 | 3 | 3 | 1 | 1 | **13** |
| 238 |  |  |  |  |  | Audio Books |  | 1 | 2 | 1 | 1 | 1 | 1 | **7** |
| 239 |  |  |  |  | Acting (Theatre) |  |  | 0 | 0 | 0 | 1 | 1 | 0 | **2** |
| 240 |  |  |  |  | Mind and Patience Games | |  | 0 | 0 | 0 | 0 | 0 | 0 | **0** |
| 241 |  |  |  |  |  | Crossword/Sudoku | | 3 | 2 | 15 | 11 | 10 | 5 | **46** |
| 242 |  |  |  |  |  | Jigsaw Puzzles |  | 0 | 0 | 0 | 0 | 1 | 0 | **1** |
| 243 |  |  |  |  |  | Memory Training |  | 0 | 0 | 1 | 1 | 1 | 1 | **4** |
| 244 |  |  |  |  | Playing Games (indoors or outdoors) | |  | 10 | 25 | 24 | 30 | 23 | 24 | **136** |
| 245 |  |  |  |  | Watching television/Streaming Videos/DVDs | | | 47 | 43 | 59 | 31 | 42 | 40 | **262** |
| 246 |  |  |  |  | Driving/Motorcycling | |  | 5 | 4 | 5 | 5 | 5 | 4 | **28** |
| 247 |  |  |  |  | Hunting/Fishing |  |  | 0 | 3 | 3 | 4 | 1 | 2 | **13** |
| 248 |  |  |  |  | Trading on the Stock Market | |  | 1 | 1 | 0 | 0 | 0 | 0 | **2** |
| 249 |  |  |  | Education |  |  |  | 2 | 0 | 7 | 2 | 3 | 6 | **20** |
| 250 |  |  |  | Sports Activities |  |  |  | 23 | 37 | 32 | 62 | 73 | 58 | **285** |
| 251 |  |  |  |  | Sports in a Group |  |  | 0 | 3 | 1 | 6 | 6 | 17 | **33** |
| 252 |  |  |  |  | Lots of Exercise |  |  | 15 | 23 | 20 | 16 | 33 | 23 | **130** |
| 253 |  |  |  |  | Gymnastics & Dancing | |  | 8 | 15 | 13 | 22 | 25 | 27 | **110** |
| 254 |  |  |  |  |  | Pilates, Qigong, Yoga | | 3 | 3 | 3 | 7 | 10 | 9 | **35** |
| 255 |  |  |  |  | Fitness Studio |  |  | 1 | 9 | 3 | 6 | 9 | 12 | **40** |
| 256 |  |  |  |  |  | Equipment Training | | 0 | 1 | 3 | 2 | 5 | 4 | **15** |
| 257 |  |  |  |  | Ergometer/Cross-Trainer | |  | 2 | 4 | 7 | 2 | 3 | 0 | **18** |
| 258 |  |  |  |  | Bicycling/Bike Touring | |  | 84 | 79 | 107 | 74 | 68 | 67 | **479** |
| 259 |  |  |  |  | (Athletic) Walking/Running | |  | 0 | 0 | 0 | 0 | 0 | 0 | **0** |
| 260 |  |  |  |  |  | Hiking |  | 44 | 62 | 85 | 80 | 69 | 57 | **397** |
| 261 |  |  |  |  |  | (Nordic) Walking |  | 26 | 34 | 36 | 26 | 31 | 21 | **174** |
| 262 |  |  |  |  |  | Running/ Jogging |  | 16 | 21 | 23 | 16 | 12 | 9 | **97** |
| 263 |  |  |  |  | Ball Sports |  |  | 0 | 0 | 0 | 0 | 0 | 0 | **0** |
| 264 |  |  |  |  |  | Soccer |  | 1 | 1 | 0 | 0 | 0 | 1 | **3** |
| 265 |  |  |  |  |  | Volleyball |  | 0 | 0 | 0 | 0 | 0 | 1 | **1** |
| 266 |  |  |  |  |  | Tennis |  | 3 | 5 | 6 | 3 | 3 | 1 | **21** |
| 267 |  |  |  |  |  | Table Tennis |  | 0 | 1 | 3 | 5 | 4 | 4 | **17** |
| 268 |  |  |  |  |  | Golf |  | 0 | 10 | 11 | 5 | 4 | 4 | **34** |
| 269 |  |  |  |  |  | Bowling |  | 0 | 2 | 0 | 0 | 2 | 1 | **5** |
| 270 |  |  |  |  | Swimming |  |  | 0 | 11 | 0 | 10 | 7 | 10 | **38** |
| 271 |  |  |  |  | Shooting Sports |  |  | 0 | 0 | 0 | 1 | 1 | 1 | **3** |
| 272 |  |  |  |  | Skiing |  |  | 1 | 0 | 1 | 1 | 2 | 0 | **5** |
| 273 |  |  |  |  | Sailing/Canoeing |  |  | 0 | 0 | 0 | 0 | 0 | 1 | **1** |
| 274 |  |  |  |  | Physically Hard work | |  | 1 | 0 | 1 | 1 | 1 | 0 | **4** |
| 275 |  |  |  | Going for a Walk |  |  |  | 309 | 327 | 358 | 287 | 259 | 220 | **1760** |
| 276 |  |  |  | Be in the Fresh Air/Outdoors/Outside | |  |  | 24 | 38 | 19 | 15 | 22 | 14 | **132** |
| 277 |  |  |  |  | Spending Time in Nature | |  | 42 | 33 | 29 | 20 | 39 | 17 | **180** |
| 278 |  |  |  |  | Spending Time in the Forest | |  | 21 | 27 | 16 | 4 | 10 | 4 | **82** |
| 279 |  |  |  | Do Something |  |  |  | 0 | 0 | 0 | 0 | 0 | 0 | **0** |
| 280 |  |  |  |  | Excursions/Activities | |  | 14 | 22 | 21 | 32 | 19 | 25 | **133** |
| 281 |  |  |  |  | Vacation/Travelling | |  | 5 | 28 | 3 | 48 | 48 | 71 | **203** |
| 282 |  |  |  |  | Stroll through the City | |  | 1 | 4 | 2 | 4 | 2 | 6 | **19** |
| 283 |  |  |  |  | Use Cultural Offers | |  | 1 | 3 | 1 | 8 | 6 | 11 | **30** |
| 284 |  |  |  |  |  | Readings |  | 0 | 0 | 0 | 0 | 0 | 3 | **3** |
| 285 |  |  |  |  |  | Library |  | 0 | 1 | 0 | 2 | 0 | 0 | **3** |
| 286 |  |  |  |  |  | Museum/Exhibition | | 2 | 3 | 1 | 3 | 8 | 5 | **22** |
| 287 |  |  |  |  |  | Concerts |  | 0 | 6 | 0 | 4 | 9 | 14 | **33** |
| 288 |  |  |  |  |  | Cinema |  | 0 | 4 | 1 | 3 | 4 | 7 | **19** |
| 289 |  |  |  |  |  | Theatre |  | 0 | 4 | 0 | 3 | 4 | 12 | **23** |
| 290 |  |  |  |  |  | Ballet |  | 0 | 1 | 0 | 0 | 0 | 0 | **1** |
| 291 |  |  |  |  |  | Opera |  | 0 | 0 | 0 | 0 | 2 | 2 | **4** |
| 292 |  |  |  |  |  | Plant Excursions |  | 1 | 0 | 0 | 0 | 0 | 0 | **1** |
| 293 |  |  |  |  | Participate in Online Events | |  | 3 | 2 | 6 | 1 | 1 | 4 | **17** |
| 294 |  |  |  |  |  | Live Streaming |  | 1 | 0 | 0 | 0 | 0 | 0 | **1** |
| 295 |  |  |  |  |  | Virtual Tours |  | 1 | 0 | 0 | 0 | 0 | 0 | **1** |
| 296 |  |  |  |  |  | Online Courses/Seminars | | 1 | 2 | 5 | 1 | 2 | 4 | **15** |
| 297 |  |  |  |  |  | Lectures on the Internet | | 1 | 1 | 2 | 0 | 2 | 0 | **6** |
| 298 |  |  |  |  | Going out for a Meal/to a Café/Beer Garden | | | 1 | 16 | 2 | 24 | 14 | 18 | **75** |
| 299 |  |  |  |  | Attend Events (not specified) | |  | 0 | 1 | 0 | 3 | 2 | 9 | **15** |
| 300 |  |  |  |  |  | Attend Speeches/Presentations (not specified) | | 0 | 0 | 0 | 0 | 1 | 1 | **2** |
| 301 |  |  |  | Use Digital Media |  |  |  | 2 | 4 | 4 | 2 | 0 | 0 | **12** |
| 302 |  |  |  |  | Apps |  |  | 2 | 0 | 1 | 0 | 0 | 1 | **4** |
| 303 |  |  |  |  | Mobile Phone/Smartphone | |  | 10 | 3 | 7 | 4 | 3 | 1 | **28** |
| 304 |  |  |  |  | Tablet/ iPad |  |  | 2 | 1 | 2 | 2 | 1 | 1 | **9** |
| 305 |  |  |  |  | PC |  |  | 8 | 11 | 11 | 6 | 3 | 4 | **43** |
| 306 |  |  |  |  | Internet |  |  | 18 | 14 | 28 | 14 | 17 | 19 | **110** |
| 307 |  |  |  | Relax & Reflect |  |  |  | 6 | 3 | 2 | 5 | 1 | 2 | **19** |
| 308 |  |  |  |  | (Day)Dreaming |  |  | 1 | 0 | 1 | 1 | 0 | 0 | **3** |
| 309 |  |  |  |  | Sauna |  |  | 1 | 0 | 0 | 2 | 4 | 1 | **8** |
| 310 |  |  |  |  | Meditation |  |  | 1 | 3 | 1 | 0 | 2 | 1 | **8** |
| 311 |  |  |  | Learn/Experience New Things | |  |  | 1 | 3 | 2 | 4 | 2 | 3 | **15** |
| 312 |  | Receiving Instrumental Social Support | |  |  |  |  | 33 | 15 | 13 | 12 | 4 | 3 | **80** |
| 313 |  |  | Cleaning Help |  |  |  |  | 1 | 2 | 0 | 0 | 0 | 0 | **3** |
| 314 |  |  | Care Service |  |  |  |  | 1 | 1 | 3 | 0 | 1 | 0 | **6** |
| 315 |  |  | Relatives live in the same House | |  |  |  | 5 | 6 | 1 | 2 | 2 | 2 | **18** |
| 316 |  |  | Get help with Errands | |  |  |  | 27 | 8 | 2 | 1 | 0 | 0 | **38** |
| 317 |  |  | Ask for Help |  |  |  |  | 0 | 0 | 0 | 1 | 0 | 0 | **1** |
| 318 |  |  | Know about possible Support from Others | | |  |  | 1 | 0 | 0 | 0 | 0 | 2 | **3** |
| 319 | Emotion-focused Strategies | |  |  |  |  |  | 0 | 0 | 0 | 0 | 0 | 0 | **0** |
| 320 |  | Maintain/Seek out Social Contacts | |  |  |  |  | 51 | 28 | 39 | 39 | 38 | 29 | **224** |
| 321 |  |  | Type/Size of Social Contacts | |  |  |  | 0 | 0 | 0 | 0 | 0 | 0 | **0** |
| 322 |  |  |  | Family |  |  |  | 96 | 103 | 143 | 138 | 112 | 144 | **736** |
| 323 |  |  |  |  | Parents |  |  | 3 | 8 | 4 | 2 | 1 | 1 | **19** |
| 324 |  |  |  |  | Parents-in-law |  |  | 1 | 1 | 0 | 2 | 0 | 0 | **4** |
| 325 |  |  |  |  | Spouse |  |  | 127 | 116 | 94 | 84 | 58 | 73 | **552** |
| 326 |  |  |  |  | Siblings |  |  | 6 | 18 | 6 | 12 | 9 | 9 | **60** |
| 327 |  |  |  |  | Own Children |  |  | 84 | 93 | 64 | 53 | 51 | 37 | **382** |
| 328 |  |  |  |  | Children-in-law |  |  | 7 | 4 | 2 | 2 | 4 | 4 | **23** |
| 329 |  |  |  |  | Grandchildren |  |  | 55 | 81 | 60 | 63 | 47 | 37 | **343** |
| 330 |  |  |  |  | Great-grandchildren | |  | 1 | 1 | 1 | 1 | 1 | 1 | **6** |
| 331 |  |  |  |  | Nephews/Nieces |  |  | 1 | 1 | 2 | 1 | 1 | 1 | **7** |
| 332 |  |  |  |  | Cousins |  |  | 0 | 1 | 0 | 1 | 0 | 0 | **2** |
| 333 |  |  |  | Friends |  |  |  | 105 | 137 | 131 | 174 | 138 | 175 | **860** |
| 334 |  |  |  | Acquaintances |  |  |  | 21 | 27 | 21 | 37 | 17 | 23 | **146** |
| 335 |  |  |  | Neighbors |  |  |  | 55 | 63 | 48 | 41 | 24 | 33 | **264** |
| 336 |  |  |  | Club/Initiative |  |  |  | 2 | 2 | 1 | 6 | 9 | 7 | **27** |
| 337 |  |  |  |  | Regulars' Table |  |  | 0 | 2 | 1 | 2 | 2 | 5 | **12** |
| 338 |  |  |  |  | Self-help Group |  |  | 2 | 2 | 2 | 2 | 1 | 1 | **10** |
| 339 |  |  |  |  | Literature Circle |  |  | 2 | 1 | 1 | 1 | 3 | 1 | **9** |
| 340 |  |  |  |  | Club |  |  | 8 | 12 | 6 | 14 | 0 | 15 | **55** |
| 341 |  |  |  |  | Choir |  |  | 3 | 0 | 1 | 6 | 2 | 8 | **20** |
| 342 |  |  |  |  | Orchestra |  |  | 0 | 0 | 0 | 1 | 2 | 4 | **7** |
| 343 |  |  |  |  | Neighborhood help | |  | 1 | 3 | 2 | 1 | 2 | 1 | **10** |
| 344 |  |  |  |  | Church community | |  | 1 | 1 | 4 | 0 | 0 | 4 | **10** |
| 345 |  |  |  |  | Retiree/Senior Meeting | |  | 0 | 1 | 0 | 2 | 2 | 2 | **7** |
| 346 |  |  |  | (Former) Professional Contacts | |  |  | 0 | 2 | 0 | 0 | 1 | 0 | **3** |
| 347 |  |  |  |  | Patients |  |  | 1 | 0 | 1 | 0 | 0 | 0 | **2** |
| 348 |  |  |  |  | Customers |  |  | 2 | 4 | 3 | 1 | 1 | 2 | **13** |
| 349 |  |  |  |  | Colleagues |  |  | 5 | 12 | 6 | 7 | 5 | 11 | **46** |
| 350 |  |  |  |  | Employers |  |  | 1 | 0 | 0 | 0 | 0 | 0 | **1** |
| 351 |  |  |  | Kids (not own Family) | |  |  | 4 | 6 | 1 | 3 | 3 | 1 | **18** |
| 352 |  |  |  | Animals |  |  |  | 0 | 0 | 0 | 0 | 0 | 1 | **1** |
| 353 |  |  |  |  | Pets |  |  | 19 | 27 | 24 | 21 | 22 | 16 | **129** |
| 354 |  |  |  |  | Animals outside |  |  | 6 | 0 | 2 | 1 | 1 | 1 | **11** |
| 355 |  |  | Social Form of Interaction | |  |  |  | 0 | 0 | 0 | 0 | 0 | 0 | **0** |
| 356 |  |  |  | Conversations (general) | |  |  | 38 | 57 | 62 | 45 | 26 | 34 | **262** |
| 357 |  |  |  | Keep in Touch |  |  |  | 0 | 1 | 1 | 2 | 2 | 1 | **7** |
| 358 |  |  |  |  | Write/Receive letters | |  | 6 | 7 | 9 | 3 | 5 | 4 | **34** |
| 359 |  |  |  |  | Send/Receive parcels/packages | |  | 1 | 0 | 0 | 1 | 0 | 0 | **2** |
| 360 |  |  |  |  | Phone Calls |  |  | 295 | 299 | 350 | 274 | 231 | 214 | **1663** |
| 361 |  |  |  |  | Personal Contact (Visits/Meetings) | |  | 93 | 117 | 109 | 163 | 120 | 131 | **733** |
| 362 |  |  |  |  |  | Eat together with Others | | 1 | 5 | 5 | 6 | 2 | 6 | **25** |
| 363 |  |  |  |  |  | Celebrations |  | 0 | 0 | 0 | 1 | 1 | 1 | **3** |
| 364 |  |  |  |  |  | Invitations |  | 1 | 1 | 3 | 4 | 2 | 2 | **13** |
| 365 |  |  |  |  | Contact online/via internet | |  | 6 | 3 | 8 | 4 | 5 | 3 | **29** |
| 366 |  |  |  |  |  | E-mail |  | 24 | 18 | 29 | 19 | 11 | 15 | **116** |
| 367 |  |  |  |  |  | Messenger (e.g. WhatsApp) | | 52 | 60 | 70 | 47 | 40 | 33 | **302** |
| 368 |  |  |  |  |  | Video calls (e.g. Zoom, Skype) | | 39 | 31 | 56 | 14 | 14 | 11 | **165** |
| 369 |  |  |  |  |  | Social media/Networks (e.g. Facebook) | | 6 | 1 | 12 | 4 | 1 | 3 | **27** |
| 370 |  |  |  |  | Communication (contact medium unknown/unspecified) | | | 13 | 8 | 7 | 5 | 5 | 1 | **39** |
| 371 |  |  |  | Get to Know New Contacts/New People | | |  | 0 | 1 | 2 | 4 | 3 | 0 | **10** |
| 372 |  |  | Receive Emotional Social Support | |  |  |  | 23 | 4 | 5 | 4 | 2 | 0 | **38** |
| 373 |  |  |  | Good Marital Relationship | |  |  | 41 | 26 | 27 | 14 | 15 | 29 | **152** |
| 374 |  |  |  | Family Cohesion/Support | |  |  | 18 | 10 | 19 | 9 | 7 | 6 | **69** |
| 375 |  |  |  | Feeling of Being Needed by Others | |  |  | 1 | 1 | 3 | 2 | 2 | 3 | **12** |
| 376 |  | Self-care |  |  |  |  |  | 3 | 4 | 3 | 2 | 1 | 0 | **13** |
| 377 |  |  | Care for Physical Well-being | |  |  |  | 1 | 1 | 0 | 0 | 0 | 1 | **3** |
| 378 |  |  |  | Sleeping |  |  |  | 4 | 2 | 1 | 1 | 0 | 1 | **9** |
| 379 |  |  |  | Enjoy good Food/Drink | |  |  | 11 | 13 | 5 | 5 | 6 | 4 | **44** |
| 380 |  |  |  |  | Drink Coffee |  |  | 1 | 1 | 2 | 1 | 0 | 0 | **5** |
| 381 |  |  |  |  | Enjoy Wine |  |  | 2 | 4 | 2 | 2 | 1 | 2 | **13** |
| 382 |  |  |  |  | Drink Beer |  |  | 1 | 0 | 0 | 0 | 0 | 0 | **1** |
| 383 |  |  |  | Special Diet (e.g. Interval Fasting) | |  |  | 0 | 1 | 0 | 1 | 1 | 0 | **3** |
| 384 |  |  |  | Healthy Eating |  |  |  | 7 | 4 | 1 | 1 | 3 | 1 | **17** |
| 385 |  |  |  | Smoking |  |  |  | 0 | 0 | 0 | 0 | 0 | 1 | **1** |
| 386 |  | Mindfulness |  |  |  |  |  | 3 | 3 | 1 | 0 | 2 | 0 | **9** |
| 387 |  |  | Diary/Journaling |  |  |  |  | 3 | 1 | 0 | 1 | 0 | 0 | **5** |
| 388 |  |  | Enjoy Season/Nature | |  |  |  | 14 | 6 | 3 | 4 | 5 | 5 | **37** |
| 389 |  |  |  | Enjoy nice Weather | |  |  | 22 | 15 | 2 | 4 | 2 | 7 | **52** |
| 390 |  |  |  | Enjoy Sunshine |  |  |  | 10 | 2 | 2 | 1 | 2 | 0 | **17** |
| 391 |  | Religion/Faith related Activities | |  |  |  |  | 0 | 0 | 0 | 1 | 0 | 0 | **1** |
| 392 |  |  | Prayer |  |  |  |  | 2 | 5 | 2 | 2 | 2 | 1 | **14** |
| 393 |  |  | Church Services |  |  |  |  | 5 | 13 | 12 | 8 | 8 | 3 | **49** |
| 394 |  |  | Community with other Believers | |  |  |  | 2 | 5 | 3 | 2 | 1 | 1 | **14** |
| 395 |  |  | Church Engagement | |  |  |  | 1 | 1 | 1 | 0 | 0 | 0 | **3** |
| 396 |  |  | Reading the Bible |  |  |  |  | 1 | 4 | 3 | 2 | 2 | 1 | **13** |
| 397 |  | Resort to unhealthy Coping Strategies | |  |  |  |  | 0 | 0 | 0 | 0 | 0 | 0 | **0** |
| 398 |  |  | Frustration Eating |  |  |  |  | 0 | 0 | 0 | 1 | 0 | 0 | **1** |
| 399 |  |  | Unhealthy Diet |  |  |  |  | 1 | 1 | 0 | 0 | 0 | 0 | **2** |
| 400 |  |  | Drink (too much) Alcohol | |  |  |  | 3 | 0 | 0 | 0 | 0 | 0 | **3** |
| 401 | Cognitive Strategies (reactive) | |  |  |  |  |  | 0 | 0 | 0 | 0 | 0 | 0 | **0** |
| 402 |  | (Re)assessment of the Situation | |  |  |  |  | 0 | 2 | 0 | 1 | 2 | 0 | **5** |
| 403 |  |  | Social Comparison/Relativization | |  |  |  | 7 | 3 | 2 | 0 | 1 | 0 | **13** |
| 404 |  | (Change) Attitude/Basic Mindset | |  |  |  |  | 0 | 1 | 1 | 0 | 0 | 0 | **2** |
| 405 |  |  | Stay Calm |  |  |  |  | 0 | 0 | 0 | 0 | 0 | 0 | **0** |
| 406 |  |  |  | Keep Calm/Composure | |  |  | 9 | 5 | 3 | 1 | 1 | 1 | **20** |
| 407 |  |  |  | Do not be Infected by Hysteria | |  |  | 11 | 9 | 3 | 3 | 2 | 5 | **33** |
| 408 |  |  | Focus on the Positive | |  |  |  | 0 | 0 | 0 | 0 | 0 | 0 | **0** |
| 409 |  |  |  | Being Satisfied with One's own Life Situation | | |  | 10 | 2 | 3 | 6 | 4 | 2 | **27** |
| 410 |  |  |  | Being Happy about Little Things | |  |  | 4 | 0 | 1 | 1 | 1 | 0 | **7** |
| 411 |  |  |  | Gratitude |  |  |  | 7 | 12 | 4 | 4 | 1 | 3 | **31** |
| 412 |  |  |  | Being Happy/Having a Good Mood | |  |  | 3 | 2 | 1 | 0 | 1 | 0 | **7** |
| 413 |  |  |  | (Beautiful) Memories | |  |  | 3 | 4 | 0 | 1 | 1 | 1 | **10** |
| 414 |  |  | Focus on Others |  |  |  |  | 0 | 0 | 0 | 0 | 0 | 0 | **0** |
| 415 |  |  |  | Bring Joy to Others | |  |  | 1 | 1 | 2 | 0 | 0 | 0 | **4** |
| 416 |  |  |  | Be There for Others | |  |  | 2 | 2 | 0 | 0 | 2 | 2 | **8** |
| 417 |  |  |  | Think of Others |  |  |  | 1 | 0 | 0 | 1 | 2 | 0 | **4** |
| 418 |  | Distraction |  |  |  |  |  | 0 | 2 | 0 | 0 | 0 | 2 | **4** |
| 419 |  | Hope |  |  |  |  |  | 1 | 2 | 0 | 1 | 6 | 2 | **12** |
| 420 |  |  | Give up Hope |  |  |  |  | 0 | 1 | 0 | 1 | 0 | 0 | **2** |
| 421 |  |  | Anticipation of the Time after the Pandemic | | |  |  | 0 | 0 | 2 | 0 | 2 | 0 | **4** |
| 422 |  |  | Hope for Normality soon | |  |  |  | 3 | 5 | 7 | 1 | 0 | 1 | **17** |
| 423 |  |  | Hope for Vaccine |  |  |  |  | 1 | 4 | 3 | 1 | 0 | 0 | **9** |
| 424 |  | Planning |  |  |  |  |  | 0 | 0 | 0 | 1 | 1 | 0 | **2** |
| 425 |  |  | Plan the Future |  |  |  |  | 3 | 4 | 2 | 2 | 1 | 0 | **12** |
| 426 |  |  | Plan a Move |  |  |  |  | 2 | 1 | 0 | 0 | 0 | 1 | **4** |
| 427 |  |  | Plan vacation/a Trip | |  |  |  | 2 | 2 | 3 | 2 | 6 | 3 | **18** |

*Note:* Data of all available records.

From a technical perspective, the categories in this table are all at the same level; therefore, they are mutually exclusive and not cumulated. All counts given apply only to the respective row and do not include counts from other rows, e.g., lower levels. However, in order to also represent the level structure generated by the qualitative content analysis, which provides important content information, the categories are visually represented at different levels.
This representation is mainly due to the fact that text fragments often had to be sorted directly into a category on a higher level without any equivalent on the level below.

**Table S3**

*Correlations Coro-Q1 (Kendall-Tau-B)*

**

**Table S4**

*Correlations Coro-Q2 (Kendall-Tau-B)*

**Table S5**

*Correlations Coro-Q3 (Kendall-Tau-B)*

|  |  | **Median** | **IQR** | **1** | **2** | **3** | **4** | **5** | **6** | **7** | **8** | **9** | **10** | **11** | **12** | **13** | **14** | **15** | **16** | **17** | **18** |
| --- | --- | --- | --- | --- | --- | --- | --- | --- | --- | --- | --- | --- | --- | --- | --- | --- | --- | --- | --- | --- | --- |
| 1. | Age | 73 | [68;77] |  |  |  |  |  |  |  |  |  |  |  |  |  |  |  |  |  |  |
| 2. | Years of Education | 14 | [12;16] | -.01 |  |  |  |  |  |  |  |  |  |  |  |  |  |  |  |  |  |
| 3 | Fear of COVID-19 | 2 | [1;4] | **-.08**** | -.05 |  |  |  |  |  |  |  |  |  |  |  |  |  |  |  |  |
| 4. | Depression | 6 | [2;11] | **.08**** | .00 | **.16**** |  |  |  |  |  |  |  |  |  |  |  |  |  |  |  |
| 5. | Perceived Stress | 12 | [7;16] | .01 | -.02 | **.19**** | **.53**** |  |  |  |  |  |  |  |  |  |  |  |  |  |  |
| 6. | Resilience | 3.7 | [3;4.2] | .00 | .03 | **-.19**** | **-.44**** | **-.54**** |  |  |  |  |  |  |  |  |  |  |  |  |  |
| 7. | Loneliness | 1 | [0;2] | -.03 | .02 | **.13**** | **.33**** | **.31**** | **-.30**** |  |  |  |  |  |  |  |  |  |  |  |  |
| 8. | Health-rel. Quality of Life | 80 | [62;85] | **-.11**** | **.08**** | **-.13**** | **-.38**** | **-.36**** | **.31**** | **-.21**** |  |  |  |  |  |  |  |  |  |  |  |
| 9. | Physical Inactivity | 3 | [2;4] | .04 | **-.08**** | .03 | **.16**** | **.15**** | **-.12**** | **.10**** | **-.18**** |  |  |  |  |  |  |  |  |  |  |
| 10. | C1: General Beliefs | 0 | [0;0] | .03 | -.04 | -.01 | **-.07*** | -.04 | .04 | -.04 | -.02 | .02 |  |  |  |  |  |  |  |  |  |
| 11. | C2: General Living Conditions | 0 | [0;0] | .03 | .01 | .01 | -.03 | -.01 | .02 | -.03 | -.02 | -.02 | .03 |  |  |  |  |  |  |  |  |
| 12. | C3: General Evaluation of the Situation | 0 | [0;0] | .02 | **-.08*** | -.01 | .03 | .05 | -.01 | .02 | -.04 | .03 | **.09*** | -.04 |  |  |  |  |  |  |  |
| 13. | C3.1: Positive | 0 | [0;0] | -.03 | .00 | .01 | .03 | .00 | .03 | .01 | .04 | .03 | .06 | -.03 | **.39**** |  |  |  |  |  |  |
| 14. | C3.2: Irrelevant | 0 | [0;0] | -.03 | -.05 | -.02 | .00 | -.02 | .01 | -.01 | -.05 | .03 | -.01 | -.05 | **.55**** | .02 |  |  |  |  |  |
| 15. | C3.3: Stressful | 0 | [0;0] | .05 | **-.07*** | .00 | .04 | **.09**** | -.05 | .03 | -.05 | .02 | **.07*** | -.03 | **.77**** | .00 | **.17**** |  |  |  |  |
| 16. | C4: Problem-focused Strategies | 2 | [1;3] | .00 | **.09**** | .05 | -.03 | -.02 | .03 | -.03 | **.08**** | **-.18**** | .00 | **.32**** | -.02 | -.01 | **-.09**** | .02 |  |  |  |
| 17. | C5: Emotion-focused Strategies | 1 | [0;3] | **-.08**** | .05 | .04 | .03 | .04 | -.02 | -.05 | .02 | .01 | .06 | **.14**** | -.01 | .03 | **-.13**** | .03 | .**28**** |  |  |
| 18. | C6: Cognitive Strategies (reactive) | 0 | [0;0] | -.01 | .01 | -.01 | -.01 | -.02 | -.01 | .02 | -.02 | -.05 | **.12**** | .05 | .10** | .07 | .03 | **.08*** | .02 | .05 |  |
| 19. | Number of Coping Strategies | 4 | [2;7] | -.04 | **.07*** | .05 | -.01 | .01 | .01 | -.04 | .04 | **-.09**** | **.10**** | **.38**** | .10** | .05 | -.05 | **.13**** | **.69**** | **.63**** | **.11**** |

*Note. N* = 796
* indicates *p* < .05, ** indicates *p* < .01
IQR = Interquartile Range [25. Percentile; 75. Percentile]

3. Fear of COVID-19: 0 (no fear) to 10 (great fear); 4. Beck Depression Inventory (BDI-II): 0-63 points; 5. Perceived Stress Scale: 0-40 points; 6. Brief Resilience Scale: 1 (low) to 5 (high); 7. 6-item-Loneliness Scale: 0 (not lonely) to 6 (very lonely) points; 8. EQ-5D-5L VAS: 0 (worst) to 100 (best); 9. Physical Inactivity: 1 (>4 hours of physical activity per week) to 5 (no physical activity)

**Table S6**

*Correlations Coro-Q4 (Kendall-Tau-B)*

**Table S7**

*Correlations Coro-Q5 (Kendall-Tau-B)*

**

**Table S8**

*Correlations Coro-Q6 (Kendall-Tau-B)*

Table S9

*Group differences between females and males in the six questionnaire rounds*

|  | **Coro-Q1**  *N* = 774  (f=367, m=407) | | **Coro-Q2**  *N* = 780  (f=377, m=403) | | **Coro-Q3**  *N* = 796  (f=380, m=416) | | **Coro-Q4**  *N* = 759  (f=364, m=395) | | **Coro-Q5**  *N* = 746  (f=354, m=392) | | **Coro-Q6**  *N* = 705  (f=333, m=372) | |
| --- | --- | --- | --- | --- | --- | --- | --- | --- | --- | --- | --- | --- |
|  | **z** | **r** | **z** | **r** | **z** | **r** | **z** | **r** | **z** | **r** | **z** | **r** |
| Age | **-3.451**** | 0.12 | **-3.150**** | 0.11 | **-3.273**** | 0.12 | **-3.739**** | 0.14 | **-4.019**** | 0.15 | **-4.013**** | 0.15 |
| Years of Education | **-7.900**** | 0.28 | **-7.595**** | 0.27 | **-8.104**** | 0.29 | **-7.782**** | 0.28 | **-7.696**** | 0.28 | **-7.274**** | 0.27 |
| Fear of COVID-19 | **3.371**** | 0.12 | **3.101**** | 0.11 | 1.810 | 0.06 | **2.315*** | 0.08 | 1.748 | 0.06 | 1.689 | 0.06 |
| Depression | **4.182**** | 0.15 | **3.983**** | 0.14 | **3.488**** | 0.12 | **3.427**** | 0.12 | **2.668**** | 0.10 | **2.507*** | 0.09 |
| Perceived Stress | **5.065**** | 0.18 | **4.943**** | 0.18 | **4.624**** | 0.16 | **4.384**** | 0.16 | **4.898**** | 0.18 | **4.216**** | 0.16 |
| Resilience | - | - | **-5.870**** | 0.21 | **-5.342**** | 0.19 | **-4.033**** | 0.15 | **-5.226**** | 0.19 | **-4.538**** | 0.17 |
| Loneliness | **3.411**** | 0.12 | **3.236**** | 0.12 | **2.758**** | 0.10 | 1.414 | 0.05 | 1.515 | 0.06 | 0.736 | 0.03 |
| Health-rel. Quality of Life | -1.224 | 0.04 | **-2.710**** | 0.10 | **-3.319**** | 0.12 | -0.349 | 0.01 | -0.856 | 0.03 | -0.845 | 0.03 |
| Physical Inactivity | 1.523 | 0.05 | **2.253*** | 0.08 | 1.433 | 0.05 | **2.592**** | 0.09 | 1.637 | 0.06 | 1.478 | 0.06 |
| C1: General Beliefs | 1.149 | 0.04 | 0.276 | 0.01 | 2.590 | 0.09 | 1.742 | 0.06 | 0.675 | 0.02 | -0.411 | 0.02 |
| C2: General Living Conditions | 1.298 | 0.05 | 0.654 | 0.02 | 1.825 | 0.06 | 0.161 | 0.01 | 1.838 | 0.07 | 1.795 | 0.07 |
| C3: General Evaluation of the Situation | **2.088*** | 0.08 | 1.759 | 0.06 | 0.206 | 0.01 | **2.019*** | 0.07 | -0.358 | 0.01 | -0.859 | 0.03 |
| C3.1: Positive | **4.600**** | 0.17 | **2.802**** | 0.10 | 0.438 | 0.02 | 1.248 | 0.05 | 0.943 | 0.03 | 0.950 | 0.04 |
| C3.2: Irrelevant | -1.114 | 0.04 | 0.154 | 0.01 | -1.296 | 0.05 | 0.789 | 0.03 | -1.325 | 0.05 | -2.160 | 0.08 |
| C3.3: Stressful | 0.585 | 0.02 | 0.702 | 0.03 | 0.492 | 0.02 | **2.082*** | 0.08 | 1.274 | 0.05 | 1.663 | 0.06 |
| C4: Problem-focused Strategies | **4.135**** | 0.15 | 1.323 | 0.05 | **2.517*** | 0.09 | 1.105 | 0.04 | **3.144**** | 0.12 | **3.591**** | 0.14 |
| C5: Emotion-focused Strategies | **6.859**** | 0.25 | **4.394**** | 0.16 | **7.761**** | 0.28 | **4.746**** | 0.17 | **6.153**** | 0.23 | **5.570**** | 0.21 |
| C6: Cognitive Strategies (reactive) | 0.331 | 0.01 | 1.499 | 0.05 | 1.165 | 0.04 | 1.450 | 0.05 | 1.476 | 0.05 | 0.683 | 0.03 |
| Total number of Coping Strategies | **5.964**** | 0.21 | **3.528**** | 0.13 | **6.053**** | 0.21 | **3.874**** | 0.14 | **5.176**** | 0.19 | **5.175**** | 0.19 |

*Note.* Mann-Whitney-U-Tests, z: z statistic, r = |z|/$\surd N$ : effect size

* indicates *p* < .05, ** indicates *p* < .01
z indicated the direction of the group difference, for z < 0 females showed lower values/scores than males, for z > 0 females showed higher values/scores than males
